# Platelet‐rich plasma (PRP) versus injectable platelet‐rich fibrin (i‐PRF): A systematic review across all fields of medicine

**DOI:** 10.1111/prd.12626

**Published:** 2025-03-24

**Authors:** Nima Farshidfar, Mohammad Amin Amiri, Nathan E. Estrin, Paras Ahmad, Anton Sculean, Yufeng Zhang, Richard J. Miron

**Affiliations:** ^1^ Department of Periodontology University of Bern Bern Switzerland; ^2^ Oral and Dental Disease Research Center, School of Dentistry Shiraz University of Medical Sciences Shiraz Iran; ^3^ Lake Erie College of Osteopathic Medicine School of Dental Medicine Bradenton Florida USA; ^4^ Department of Research Advanced PRF Education Jupiter Florida USA; ^5^ Department of Oral Implantology University of Wuhan Wuhan China

**Keywords:** bio‐PRF, I‐PRF, L‐PRF, platelet‐rich fibrin, platelet‐rich plasma

## Abstract

This systematic review aimed to evaluate all available evidence across all fields of medicine regarding the comparative effectiveness of platelet‐rich plasma (PRP) versus injectable platelet‐rich fibrin (i‐PRF). A comprehensive search was conducted in PubMed, Scopus, and Web of Science up to September 30, 2024. Following a thorough screening process, studies were divided into in vitro, in vivo, and clinical studies based on their tissue/clinical indications. The initial search generated 2192 articles, of which 23 met the inclusion criteria. The findings demonstrated that i‐PRF yielded higher platelet concentrations and offered a more sustained, long‐term release of growth factors over time when compared to PRP. Overall, it was determined from in vitro studies that i‐PRF significantly improved the activity of many cell types, including for skin, cartilage, periodontal, bone, soft tissue around dental implants, and pulp cells. In vivo outcomes also generally indicated that i‐PRF outperformed PRP in cartilage and testicular regeneration. However, in orthodontic tooth movement, PRP was found to lead to superior short‐term effects, while i‐PRF showed more beneficial long‐term effects. Clinical studies also found superior outcomes of i‐PRF in skin regeneration, cartilage, and pulp regeneration. Outcomes regarding orthodontic tooth movement utilizing i‐PRF or PRP remain controversial. In 72% of studies, i‐PRF was found to lead to better outcomes across the various fields of medicine when compared to PRP, whereas 24% found no differences between the groups. Reasons and inconsistencies across the studies may be attributed to protocol differences and tissue types. Overall, additional clinical studies are needed with well‐designed research and centrifugation protocols to better understand the regenerative potential of platelet concentrates in medicine. i‐PRF offers a more sustained and prolonged release of growth factors and was favored in the majority of studies over PRP and should, therefore, be favored for the majority of medical and dental applications.

## 1 INTRODUCTION

Autologous platelet concentrates (APCs) are increasingly popular biomaterials used in various fields of medicine owing to their ability to release supra‐concentrates of autologous growth factors, including transforming growth factor‐beta (TGF‐β), vascular endothelial growth factor (VEGF), and platelet‐derived growth factor (PDGF).^1^, ^2^, ^3^, ^4^, ^5^, ^6^, ^7^ The first generation of APCs was developed in the late 1990s and termed platelet‐rich plasma (PRP), a liquid form of platelet concentrates utilizing anticoagulants.^8^, ^9^ PRP has successfully been utilized in various clinical applications, including wound healing, skin rejuvenation, hair restoration, orthopedic surgeries, and dental procedures.^10^, ^11^, ^12^ Protocols for PRP include tubes containing anticoagulants, which have since been hypothesized to interfere with the natural clotting process, potentially hindering tissue regeneration in the affected area.^13^, ^14^ Since all wound healing begins with clot formation, the presence of anticoagulants has been hypothesized to impair wound healing and the prolonged release of growth factors over time.^15^, ^16^ Studies have demonstrated that PRP exhibits a rapid initial burst of growth factors released shortly after application, followed by a decreased release rate over time.^15^ This release pattern may, however, not be ideal for sustained tissue regeneration.

To address these limitations, platelet‐rich fibrin (PRF) was introduced as a second‐generation platelet concentrate with the main aim of anticoagulant removal.^17^, ^18^, ^19^ PRF, therefore, produces a clot following centrifugation and is capable of more slowly releasing its growth factor content over time as would be found during natural intrinsic healing.^15^, ^20^, ^21^ Obtaining this type of platelet concentrate, however, requires prompt handling, as the coagulation process begins almost immediately upon the blood sample's contact with the tube walls.^22^ The centrifugation process for PRF is a single‐step procedure in which a fibrin clot is formed in the “platelet‐rich” layer containing leukocytes embedded in the fibrin matrix in addition to platelets, growth factors, and cytokines.^23^, ^24^ While various comparative studies have investigated PRF versus PRP, one of the main advantages of PRP over the years has been its ability to remain liquid over time allowing it to be utilized as an injectable APC in many clinical scenarios.

With the introduction of the low‐speed centrifugation concept (LSCC) for developing and optimizing PRF,^25^, ^26^ injectable platelet‐rich fibrin (i‐PRF) was introduced as a liquid form of PRF with shorter centrifugation protocols.^27^, ^28^, ^29^, ^30^, ^31^ According to the LSCC, it was hypothesized that a slower, shorter centrifugation process could result in a liquid fraction of PRF, essentially liquid fibrinogen and thrombin that had not yet converted to a fibrin clot.^25^, ^26^ Additionally, the use of plastic hydrophobic tubes and an adjusted centrifugation settings (60 g for 3 min) during i‐PRF preparation favored its liquid formation. Following injection, however, the i‐PRF gradually clots, becoming a fibrin mesh with entrapment of growth factors that may continuously be released over the next 10–14 days. This sustained release creates a favorable microenvironment that promotes cellular recruitment, proliferation, and differentiation, all of which are critical for tissue regeneration.^27^, ^28^, ^29^, ^30^, ^31^

To date, the clinical use of both PRP and i‐PRF has been extensively researched, demonstrating effectiveness in various fields of regenerative medicine and dentistry. Due to their fluid nature, both PRP and i‐PRF can be easily injected into the target tissues or combined with other biomaterials to enhance their regenerative potential.^32^, ^33^, ^34^ In recent years, numerous studies in medicine have been conducted with the goal of comparing the effects of these platelet concentrates across different settings, aiming to determine which APC is most effective.

Given this substantial body of research comparing i‐PRF to PRP across various fields of medicine, a thorough assessment of the current evidence is needed to compare clinical outcomes. Therefore, this systematic review aims to evaluate all available studies regarding the comparative effectiveness of i‐PRF versus PRP in in vitro, in vivo, and clinical studies across all fields of medicine. Furthermore, by discussing the limitations of existing research and proposing new directions for future studies, this review seeks to help clinicians and researchers better understand the potential of both types of APCs, ultimately enhancing their application in clinical practice.

## 2 MATERIALS AND METHODS

### 2.1 Protocol development

The protocol followed in this systematic review was adopted from the 2020 Preferred Reporting Items for Systematic Reviews and Meta‐Analyses (PRISMA) guideline.^35^

### 2.2 Focused question

When comparing PRP and i‐PRF, which shows better outcomes in terms of platelet and growth factor concentrations, cell and tissue regeneration, microbial activity, and clinical effectiveness across all fields of medicine?

### 2.3 Eligibility criteria

The inclusion and exclusion criteria were established according to the Participants, Intervention, Comparison, Outcome, and Study design (PICOS) framework, as outlined in Table 1. In summary, this study included all in vitro, in vivo, and clinical studies that compared the use of i‐PRF with PRP across various fields of regenerative medicine. Studies published in languages other than English were excluded from our review due to the linguistic proficiency of the research team.

**TABLE 1:** Eligibility criteria for the present systematic review.

Domains	Inclusion criteria	Exclusion criteria
Participants	Studies examining platelet counts and growth factor release Studies involving cells, microorganisms Studies involving animal models Studies involving patients	–
Intervention	Use of fluid, liquid, or injectable Platelet‐rich fibrin (i‐PRF)	Studies using other PRF types (e.g., L‐PRF, A‐PRF) without i‐PRF data
Comparison	Use of platelet‐rich plasma (PRP)	Studies without a PRP comparison Studies comparing i‐PRF with non‐PRP alternatives
Outcome	Any outcomes related to platelet counts and growth factor release profiles Any outcomes related to the response of cells, microorganisms, and tissues following treatment with i‐PRF and PRP	–
Study design	In vitro, in vivo, and clinical studies	Editorials, expert opinions, conference abstracts, narrative and systematic reviews

### 2.4 Databases and search strategy

An electronic search was conducted using the PubMed, Scopus, and Web of Science databases with the following keywords applied to the title and abstract of the published studies:

((Platelet Rich Fibrin) OR (Platelet‐rich Fibrin) OR (PRF)) AND ((Platelet Rich Plasma) OR (Platelet‐rich Plasma) OR (PRP))

Articles published up to September 30, 2024, were included in this study. The reference list of the eligible included studies and other relevant reviews were also screened for possible inclusion of additional relevant studies.

### 2.5 Study selection process

The titles and abstracts of the retrieved articles were independently screened by two reviewers (NF and MAA) according to the inclusion criteria. Any discrepancies were resolved through discussion among all authors. The full texts of the selected abstracts were then obtained, and the screening process was conducted independently by the two reviewers (NF and MAA). Subsequently, articles meeting the inclusion criteria were processed for data collection.

### 2.6 Data collection

Data collection was performed according to the inclusion criteria. A custom‐made data collection form was developed to gather information from the included studies. The data collected included the authors' names, year of publication, study type and aim, characteristics of the cells, microorganisms, animals, or humans, preparation methods of i‐PRF and PRP, and details of the interventions, methods, and outcome measures. This information was then summarized in the tables presented in the results section.

## 3 RESULTS

### 3.1 Study selection

**FIGURE 1:**
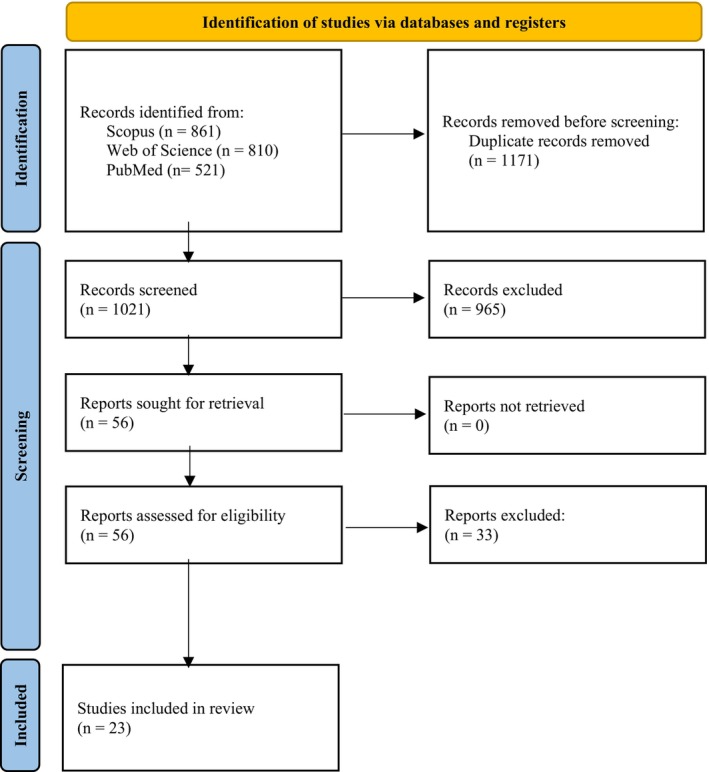
PRISMA 2020 flowchart for study selection.

In total, the initial search strategies generated 2192 articles. After duplicate removal, 1021 articles remained for title and abstract evaluation. A total of 965 papers were excluded due to a mismatch with our search criteria, and 56 articles were retained for the final full‐text review. Finally, 23 papers were included in this systematic review (Figure 1).

### 3.2 Study characteristics

In our systematic review, we categorized the included studies into in vitro, in vivo, and clinical studies. Of the 24 studies, 11 were exclusively in vitro,^31^, ^36^, ^37^, ^38^, ^39^, ^40^, ^41^, ^42^, ^43^, ^44^, ^45^ two were exclusively in vivo,^46^, ^47^ and eight were clinical^48^, ^49^, ^50^, ^51^, ^52^, ^53^, ^54^, ^55^ studies. Additionally, two studies included both in vitro and in vivo results,^56^, ^57^ which were classified under both categories, leading to a total of 13 studies in the in vitro section^31^, ^36^, ^37^, ^38^, ^39^, ^40^, ^41^, ^42^, ^43^, ^44^, ^45^, ^56^, ^57^ and 4 in the in vivo section.^46^, ^47^, ^56^, ^57^

**FIGURE 2:**
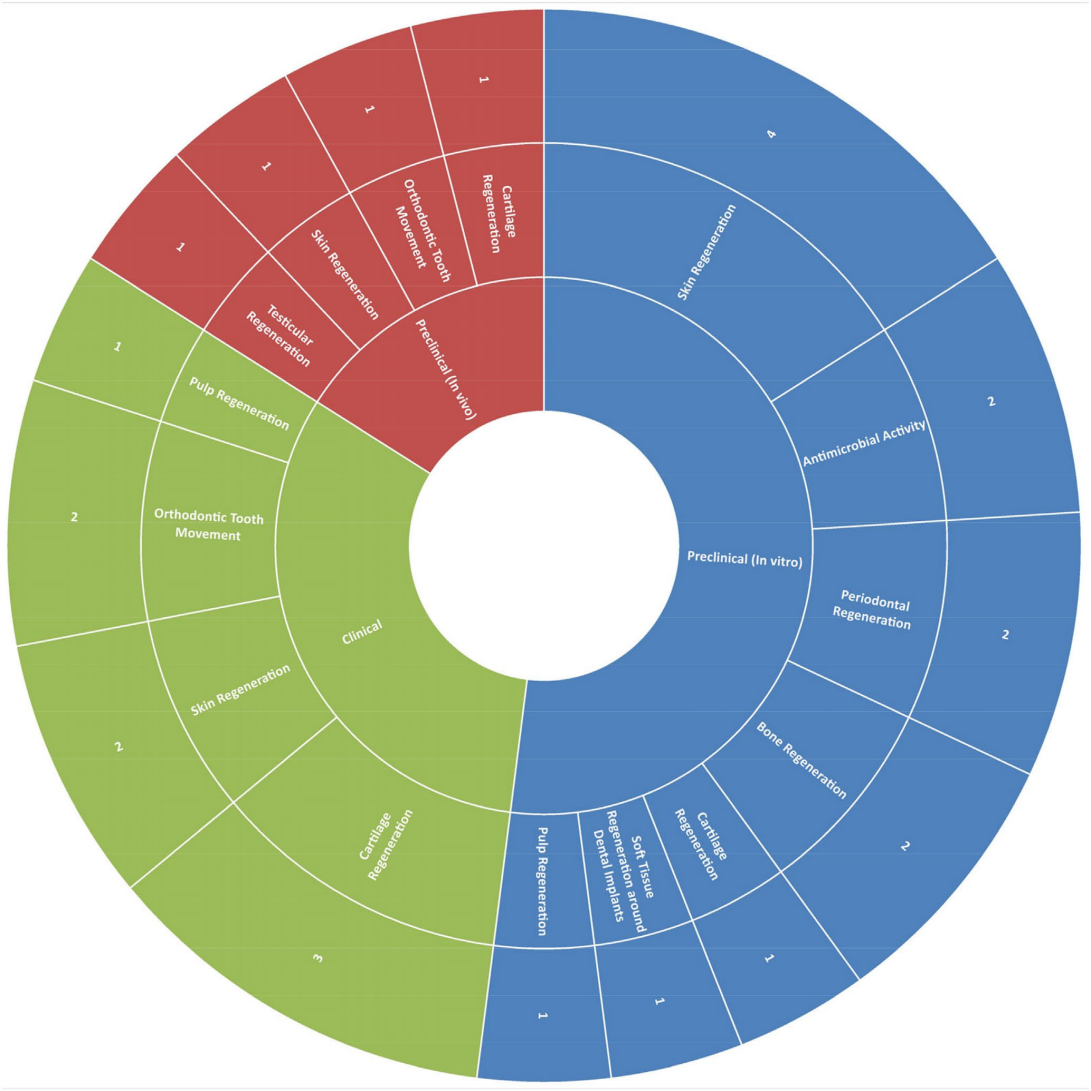
Schematic overview of the included studies based on their settings. Studies that reported both in vitro and in vivo results are presented separately in the graph.

Figure 2 provides a schematic representation of the number of studies across these settings and various fields of medicine. Among the 13 in vitro studies,^31^, ^36^, ^37^, ^38^, ^39^, ^40^, ^41^, ^42^, ^43^, ^44^, ^45^, ^56^, ^57^ four focused on skin regeneration,^40^, ^41^, ^44^, ^57^ two on periodontal regeneration,^31^, ^45^ one on soft tissue regeneration around dental implants,^42^ two on bone regeneration,^37^, ^43^ two on antimicrobial activity,^38^, ^39^ one on cartilage regeneration,^56^ and one on pulp regeneration.^36^ Of the four in vivo studies,^46^, ^47^, ^56^, ^57^ one addressed cartilage regeneration,^56^ one focused on orthodontic tooth movement (OTM),^46^ one on skin regeneration,^57^ and one on testicular regeneration.^47^ In the clinical setting,^48^, ^49^, ^50^, ^51^, ^52^, ^53^, ^54^, ^55^ two studies focused on skin regeneration,^51^, ^52^ three studies on cartilage regeneration,^49^, ^54^, ^55^ followed by two studies on OTM,^50^, ^53^ and one study on pulp regeneration.^48^ All study characteristics are also summarized in Tables 2 and 3. In Table 4, we provide a summary of the study outcomes, indicating which of PRP or i‐PRF showed an overall superior performance.

**TABLE 2:** Summary of the included preclinical studies (in vitro and in vivo).

Preclinical studies (in vitro and in vivo)								
Authors (Year)	Category	Aim of study	Cells (Source) or Animal model	Blood source	Preparation method of PRP (Device)	Preparation method of i‐PRF (Device)	Groups	Main methods and results
In Vitro Studies								
Chai et al. (2019)^36^	Pulp regeneration	To compare the cellular regenerative activity of human dental pulp cells when cultured with either i‐PRF or PRP	Dental pulp cells (Human)	Human—Healthy individuals (20–40 years old)	Anticoagulant Agent: EDTA (1) 900 g 5 min (2) 2000 g 15 min (NR)	700 rpm 3 min (NR)	(1) Control (2) PRP (3) i‐PRF	*Migration by Scratch Wound Healing and Transwell Assay (At 24 h), and Proliferation by CCK‐8 Assay (On 1st, 3rd and 5th Day)*: Both PRP and i‐PRF enhanced the migration and proliferation of human dental pulp cells compared to the control group, with i‐PRF demonstrating a significantly greater increase in both migration and proliferation than PRP. *Mineralization by ALP Assay (On 7th Day), Alizarin Red Staining (On 14th Day), and Odontogenic‐related Gene Expression by RT‐PCR (On 14th Day)*: i‐PRF significantly increased ALP activity, alizarin red staining, and the mRNA expression of genes encoding COL1a1, DSPP, and DMP‐1 compared to PRP.
Fernández‐Medina et al. (2019)^37^	Bone regeneration	To investigate the composition and bioactivity of four common clinical‐grade hemoderivates (A‐PRF, i‐PRF, P‐PRP, and L‐PRP) prepared using standardized methods	Osteoblasts (Human)	Animal—Merino sheep (3–4 years old)	Anticoagulant Agent: Sodium Citrate L‐PRP: (1) 2400 rpm (708 g) 10 min (2) 3600 rpm (1594 g) 15 min (Duo Centrifuge, Australia) Activation Agent: 10% Calcium Chloride P‐PRP: Anticoagulant Agent: Sodium Citrate (1) 1800 rpm (398 g) 8 min (Duo Centrifuge, Australia) Activation Agent: 10% Calcium Chloride	700 rpm (60 g) 3 min (Duo Centrifuge, Australia)	(1) Control (2) Blood Clot (3) A‐PRF (4) i‐PRF (5) P‐PRP (6) L‐PRP	*Platelet Count*: A‐PRF (307 ± 14 × 10^3^) and i‐PRF (336 ± 18 × 10^3^) had platelet counts comparable to those of whole blood (362 ± 22 × 10^3^) and natural clot (362 ± 32 × 10^3^). While P‐PRP demonstrated a three‐fold higher platelet recovery (934 ± 32 × 10^3^) compared to whole blood, L‐PRP exhibited the highest platelet concentration (1.82 ± 17 × 10^6^). *Growth Factors Release by ELISA (On 1st, 3rd, 7th and 14th Days)*: A significant cumulative release of IGF‐I and PDGF‐BB was noted for A‐PRF and L‐PRP groups at early time points whereas similar release profiles of BMP‐2 and VEGF were noted in all protocols. *Human Osteoblasts Viability by Live/Dead Assay (At 24 and 72 h), and Migration by Scratch Assay (At 6 and 24 h)*: Cell viability and migration assay showed detrimental effect when the concentration of all groups was ≥60%. *Metabolic Activity by Alamar Blue Assay (At 24 and 72 h)*: L‐ PRP and P‐PRP promoted higher metabolic activity than the control group at concentrations above 80% on the first day. In contrast, i‐PRF exhibited reduced metabolic activity at concentrations above 80% compared to L‐PRP and P‐PRP on the same day. However, by the third day, i‐PRF was comparable to the other platelet concentrates at concentrations greater than 40%. *Human Osteoblast Mineralization by Alizarin Red Staining (On 14th, and 21st Day)*: At 21st Day, superior mineralization properties for i‐PRF was achieved compared to all groups.
Wang et al. (2017)^43^		To investigate the effect of i‐PRF on osteoblast behavior compared to PRP	Osteoblasts (Human)	Human—Researchers within the laboratory	Anticoagulant Agent: EDTA (1) 900 g 5 min (2) 2000 g 15 min (NR)	700 rpm 3 min (Duo Centrifuge, France)	(1) Control (2) PRP (3) i‐PRF	*Viability by Live/Dead Assay (At 24 h)*: All cells had high survival rates throughout the entire study period irrespective of culture‐condition. *Migration by Transwell Assay (At 24 h), Adhesion by DAPI Staining (At 2, 4 and 8 h), and Proliferation by CCK‐8 Assay (On 1st, 3rd and 5th Day)*: While PRP induced a significant two‐fold increase in osteoblast migration, i‐PRF demonstrated a three‐fold increase compared to the control and PRP groups. Although no differences were observed in cell attachment, i‐PRF resulted in a significantly higher proliferation rate at three and five days compared to PRP. *Mineralization by ALP Assay (On 7th Day) and Alizarin Red Staining (On 14th Day)*: i‐PRF induced significantly greater ALP staining at 7 days and alizarin red staining at 14 days. *Osteogenic‐related Gene Expression by RT‐PCR on (3rd and 14th Day) and Immunofluorescent Staining of Osteocalcin (on 14th Day)*: A significant increase in mRNA levels of ALP, Runx2, and osteocalcin, along with immunofluorescent staining of osteocalcin, was also observed in the i‐PRF group compared to PRP.
Zheng et al. (2020)^45^	Periodontal regeneration	To evaluate the biological effect of PRP and I‐PRF on human periodontal ligament cells in vitro	Periodontal ligament cells (Human)	Human—Volunteer donors	Anticoagulant Agent: EDTA (1) 900 g 5 min (2) 2000 g 15 min (NR)	700 rpm 3 min (IntraSpin, USA)	(1) Control (2) PRP (3) i‐PRF	*Proliferation by CCK‐8 Assay (On 1st, 3rd and 5th Day), and Migration by Transwell and Scratch Wound Healing Assay (At 24 h)*: Human periodontal ligament cell proliferation was enhanced by both PRP and i‐PRF on days 3 and 5, with i‐PRF showing significantly higher proliferation compared to all other groups. Additionally, human periodontal ligament cells demonstrated greater migratory ability when incubated with i‐PRF compared to PRP. *Mineralization by ALP Assay (On 7th Day) and Alizarin Red Staining (On 14th Day)*: PRF significantly induced greater ALP activity compared to both the control and PRP groups. Additionally, human periodontal ligament cells incubated with i‐PRF formed more mineralized nodules. *Osteogenic‐related Gene Expression by RT‐PCR (on 14th Day)*: All osteogenic markers were up‐regulated by PRP and i‐PRF and exhibited the highest levels with liquid PRF group when compared with all groups.
Miron et al. (2017)^31^		To compare i‐PRF to the clinically utilized PRP for total growth factor release as well as on human gingival fibroblast cell biocompatibility and activity	Gingival fibroblasts (Human)	Human—6 volunteer donors	Anticoagulant Agent: Acid Citrate Dextrose (1) 1000 rpm (123 g) 7 min (2) 3000 rpm (1107 g) 10 min (Duo Centrifuge, France)	700 rpm (60 g) 3 min (Duo Centrifuge, France)	(1) Control (2) PRP (3) i‐PRF	*Growth Factors Release by ELISA (At 15 min, 60 min, 8 h, and On 1st, 3rd, and 10th Day)*: PRP exhibited a higher early release of growth factors, while i‐PRF showed significantly greater total long‐term release of PDGF‐AA, PDGF‐AB, EGF, and IGF‐1 after 10 days. Additionally, PRP demonstrated higher levels of TGF‐β1 and VEGF at the 10‐day mark. *Viability by Live/Dead Assay (At 24 h), Migration by Transwell Assay (At 24 h), and Proliferation by MTS Colorimetric Assay (On 1st, 3rd, and 5th Day)*: While both formulations exhibited high biocompatibility and enhanced fibroblast migration and proliferation compared to control tissue culture plastic, i‐PRF induced significantly greater migration, whereas PRP demonstrated significantly higher cellular proliferation. *Gene Expression by RT‐PCR on (3rd and 7th Day)*: i‐PRF showed significantly highest mRNA levels of TGF‐β at 7 days, PDGF at 3 days, and collagen1 expression at both 3 and 7 days when compared to PRP.
Wang et al. (2017)^42^	Soft tissue regeneration around dental implants	To evaluate the effect of i‐PRF on human gingival fibroblasts cultured on smooth and roughened titanium implant surfaces compared to PRP	Gingival fibroblasts (Human)	Human—Laboratory members (25–45 years old)	Anticoagulant Agent: EDTA (1) 900 g 5 min (2) 2000 g 15 min (NR)	700 rpm 3 min (Duo Centrifuge, France)	(1) TCP (Control) (2) TCP + PRP (3) TCP + i‐PRF (4) PT (Control) (5) PT + PRP (6) PT + i‐PRF (7) SLA (Control) (8) SLA + PRP (9) SLA + i‐PRF	*Viability by Live/Dead Assay (At 24 h)*: Both PRP and I‐PRF demonstrated excellent cell viability and biocompatibility. *Migration by Transwell Assay (At 24 h)*: Both PRP and i‐PRF promoted cell migration at 24 h. PRP induced a significant 250% increase in migrated cells relative to controls, while i‐PRF resulted in a greater than 350% significant increase compared to controls. *Proliferation by CCK‐8 Assay (On 1st, 3rd and 5th Day)*: On the first day, no significant differences were observed among the groups. However, on the third and fifth days, both PRP and i‐PRF significantly increased cell numbers compared to their respective controls, with i‐PRF showing significantly higher cell counts than all other groups. *Adhesion by DAPI Staining (At 2, 4, 8 h)*: No significant difference was observed between all groups. *Morphology by Phalloidin‐FITC and DAPI Staining (At 8 h)*: Both PRP and i‐PRF promoted the spreading of human gingival fibroblasts on TCP and PT compared to their respective controls, while cells seeded on SLA surfaces exhibited reduced spreading across all groups. Analysis of cell surface area showed that both PRP and i‐PRF increased the cell surface area on TCP and PT, but not on SLA.
								*Regeneration‐ and ECM‐related Gene Expression by RT‐PCR (On 7th Day)*: i‐PRF demonstrated significantly higher PDGF and TGF‐β mRNA levels across all surfaces, with no differences observed between the groups. Additionally, both PRP and i‐PRF induced an increase in COL1 and FN1 mRNA levels compared to their respective controls, with i‐PRF showing significantly higher results. *Immunofluorescent Staining of COL1 (On 7th Day)*: PRP exhibited a slight increase in immunofluorescent staining of COL1 when compared to their respective controls, while i‐PRF demonstrated the greatest fluorescence intensity in COL1 expression on all surfaces.
Abd El Raouf et al. (2019)^56^	Cartilage regeneration	To evaluate the effect of i‐PRF on cultivated chondrocytes and osteochondral regeneration in critical‐sized osteochondral defect of the rabbit's knee in comparison with PRP	Chondrocytes (Rabbit)	Animal—5 adult rabbits	Anticoagulant Agent: Sodium Citrate (1) 900 g 5 min (2) 2000 g 15 min (NR)	700 rpm (60 g) 3 min (Duo Centrifuge, France)	(1) Control (2) PRP (3) i‐PRF	*Proliferation by CCK‐8 Assay (On 1st, 3rd, 5th, and 7th Day), and Chondrogenic‐related Gene Expression by RT‐PCR (On 7th Day)*: i‐PRF significantly promoted chondrocyte proliferation and mRNA levels of SOX9, COL2A1, and ACAN when compared to PRP and control. *Inflammatory and Chondrogenic‐related Gene Expression in an Inflammatory Environment by RT‐PCR (On 7th Day)*: Both PRP and i‐PRF upregulated the expression levels of pro‐regenerative genes including SOX9, COL2A1 and ACAN and downregulated the expression levels of ADAMTS4, PTGS2 and MMP13; however, i‐PRF demonstrated the highest expression of SOX9, COL2A1 and ACAN and the lowest expression of ADAMTS4 and PTGS2.
Kour et al. (2018)^39^	Antimicrobial activity	To evaluate the antimicrobial effect of i‐PRF against two periodontal pathogens compared with PRP and PRF	*Porphyromonas gingivalis* and *Aggregatibacter actinomycetemcomitans*	Human—10 systematically and periodontally healthy Individuals (>25 years old)	Anticoagulant Agent: 3.2% Sodium Citrate (1) 1000 rpm 13 min (2) 2000 rpm 10 min (NR)	700 rpm 3 min (NR)	(1) PRP (2) PRF (3) i‐PRF	*Antimicrobial Assay by Disk Diffusion Method*: In the case of *Porphyromonas gingivalis*, i‐PRF exhibited the widest zone of inhibition, which was significantly greater than that of PRF. Additionally, PRP demonstrated a significantly wider zone of inhibition compared to PRF. For *Aggregatibacter actinomycetemcomitans*, PRP showed a wider zone of inhibition, significantly larger than that of both PRF and i‐PRF.
Karde et al. (2017)^38^		To evaluate the antimicrobial effect and platelet count of i‐PRF compared with PRP, PRF, and whole blood	Supragingival plaque samples of individuals who volunteered for blood sample.	Human—10 patients with chronic generalized marginal gingivitis (32 ± 5.6 years old)	Anticoagulant Agent: 3.2% Sodium citrate (1) 1000 rpm 10 min (2) 2000 rpm 20 min (NR)	700 rpm 3–4 min (NR)	(1) Whole blood (2) PRP (3) PRF (4) i‐PRF	*Platelet Count*: i‐PRF showed statistically significant difference in platelet count when compared to control. It was also significant when compared to PRP and PRF. *Antibacterial Assay by Disk Diffusion Method*: The mean zone of inhibition around i‐PRF and PRF reached statistical significance. Although a distinct zone of inhibition was observed with PRP, it was not statistically significant.
Xu et al. (2024)^44^	Skin Regeneration	To compare the effect of i‐PRF and PRP on trichogenic ability of dermal papilla cells	Dermal papilla cells (Mouse and Human)	Human—Healthy volunteers	Pro health care system Prosys PRP Kit – KMedicins, Korea (NR)	‐ (Sorvall ST1 Plus desk centrifuge, ThermoFisher Scientific)	(1) Control (2) PRP (3) i‐PRF	*Proliferation by CCK‐8 Assay (At Baseline, 24, 72, 120 h), and Migration by Transwell Assay (At 24 h)*: Both i‐PRF and PRP enhanced the viability, proliferation, and migration of dermal papilla cells; however, i‐PRF had a significantly greater impact on promoting these capacities compared to PRP. *Gene Expression by RT‐PCR, Protein Expression by Western Blot, and Immunofluorescence Staining Assay (On 14th Day)*: Both PRP and i‐PRF can enhance the expression of ALP, Versican, and β‐catenin; however, i‐PRF induced significantly higher levels of these proteins. Additionally, both PRP and i‐PRF stimulated the TGF‐β/Smad pathway, with i‐PRF demonstrating a more pronounced effect than PRP.
Li et al. (2022)^40^		To compare the therapeutic effects of i‐PRF and PRP on UVA‐induced photo‐aging in human dermal fibroblasts	Dermal fibroblasts (Human)	Human—4 healthy female laboratory postgraduate students (20–30 years old)	Anticoagulant Agent: Sodium Citrate (1) 900 g 10 min (2) 2000 g 15 min (NR) Activation 10% Agent: Calcium Chloride	60 g 3 min (NR)	(1) Control (2) UVA (3) UVA + PRP (4) UVA + i‐PRF	*Proliferation by CCK‐8 Assay (On 1st, 2nd, 3rd Day), and Migration by Transwell Assay (At 6 h)*: i‐PRF was more effective than PRP in promoting cell proliferation and migration. *ROS Levels by DCF‐DA Assay (After 1 h) and Cell Apoptosis by Annexin V/PI Staining and Flow Cytometry (After 24 h)*: i‐PRF reduced ROS generation and cell apoptosis more effectively than PRP. *Protein Expression by Western Blot (After 24 h)*: In terms of COL1 upregulation mechanisms, i‐PRF demonstrated a more potent stimulation of the TGF‐β/Smad signaling pathway and a greater suppression of MMP‐1 expression compared to PRP.
Tian et al. (2022)^57^		To compare the repair effects of i‐PRF prepared by a low‐speed short‐time protocol and PRP prepared by secondary centrifugation method on radiation‐induced skin injury models	Umbilical vein endothelial cells and dermal fibroblasts (Human)	Human—10 healthy volunteers	(1) 1000 rpm (123 g) 7 min (2) 3000 rpm (1107 g) 10 min (NR) Activation Agent: 10% Calcium Chloride	700 rpm (60 g) 3 min	(1) Control (2) PRP (3) i‐PRF and (1) Control (2) 10 Gy (3) 10 Gy + PRP (4) 10 Gy + i‐PRF	*Platelet Count, and Growth Factors Release by ELISA (On 3rd Day)*: Both PRP and i‐PRF exhibited significantly higher platelet counts compared to whole blood. Furthermore, i‐PRF contained a notably greater number of platelets than PRP. After 3 days, PRP demonstrated a higher release of TGF‐β1 and VEGF, while i‐PRF showed an increased release of EGF and IGF‐1. *Proliferation by CCK‐8 Assay (After 12, 24, 36, and 48 h)*: At 48 h, cell viability was 125.54%, 136.29%, and 145.47% in the non‐radiation control, PRP, and i‐PRF groups, respectively. In the radiation groups, cell viability was 125.54%, 60.29%, 52.47%, and 81.72% in the control, 10 Gy, 10 Gy + PRP, and 10 Gy + i‐PRF groups, respectively *Migration by Scratch Wound Healing Assay (After 12 h)*: The cell migration rates were 7.02%, 34.96%, and 53.05% respectively, in control group, 10 Gy + PRP group and 10 Gy + i‐PRF group. *Angiogenesis by Tube Formation Assay (After 12 h)*: After 12 h of culture, vascular endothelial cells exposed to ionizing radiation in the experimental groups demonstrated a significantly reduced tube‐forming capacity compared to the control group. In contrast, both PRP and i‐PRF enhanced tube‐forming capacity, surpassing even the levels observed in the control group. *Cell Apoptosis by Annexin V‐FTIC Staining and Flow Cytometry (After 24 h), and ROS Levels by DCF‐DA Staining and Flow Cytometry (After 24 h)*: After 24 h, the ROS levels were 1.83 × 10^6^, 5.03 × 10^6^, 2.92 × 10^6^ and 2.76 × 10^6^, respectively, in control group, 10 Gy group, 10 Gy + PRP group and 10 Gy + i‐PRF group. Additionally, the apoptosis rates in experimental groups were 8.89%, 25.91%, 22.94% and 17.87%, respectively, following the same order.
Wang et al. (2019)^41^		To compare i‐PRF to PRP on skin cell behavior and regeneration.	Dermal fibroblasts (Human)	Human—Volunteer laboratory members	Anticoagulant Agent: EDTA (1) 900 g 5 min (2) 2000 g 15 min (NR)	700 rpm 3 min (NR)	(1) Control (2) PRP (3) i‐PRF	*Viability by Live/Dead Assay (At 24 h)*: All platelet concentrates were non‐toxic to cells demonstrating high cell survival. *Migration by Transwell Assay (At 24 h)*: Skin fibroblasts migrated over 350% more in i‐PRF when compared to control and PRP with 200% increase. *Proliferation by CCK‐8 Assay (On 1st, 3rd and 5th Day)*: i‐PRF significantly induced greater cell proliferation at 5 days. *Morphology Assay by Phalloidin‐FITC and DAPI Staining (At 2,4, 8, and 24 h)*: Both PRP and i‐PRF enhanced skin fibroblast spreading at 2, 4, and 8 h; however, no significant difference was noted at 24 h. *Regeneration‐ and ECM‐related Gene Expression by RT‐PCR (On 3rd and 7th Day)*: While both PRP and i‐PRF significantly elevated cell mRNA levels of PDGF, the i‐PRF group exhibited the highest levels of TGF‐beta, COL1, and FN1 mRNA. *Immunofluorescent Staining of COL1 (On 7th Day)*: i‐PRF demonstrated a significantly greater ability to induce collagen matrix synthesis when compared to PRP.
In vivo studies								
Abd El Raouf et al. (2019)^56^	Cartilage regeneration	To evaluate the effect of i‐PRF on cultivated chondrocytes and osteochondral regeneration in critical‐sized osteochondral defect of the rabbit's knee in comparison with PRP	12 adult female New Zealand White rabbits	Animal—5 adult rabbits	Anticoagulant Agent: Sodium citrate (1) 900 g 5 min (2) 2000 g 15 min (NR)	700 rpm (60 g) 3 min (Duo Centrifuge, France)	(1) Control (2) PRP (3) i‐PRF	*Macroscopic and Microscopic Evaluation of Cartilage Defect by ICRS Scoring System (At 4th and 12th Week)*: At 4 weeks, the macroscopic ICRS scores for the i‐PRF group were significantly higher than those of the PRP and control groups. By 12 weeks post‐surgery, the microscopic ICRS scores indicated that the i‐PRF group showed a significant improvement in cartilage regeneration compared to the PRP group.
Alaa et al. (2023)^46^	Orthodontic tooth movement	To compare between the effects of submucosal injection of i‐PRF versus PRP on orthodontic tooth movement in a rabbit model over a 28‐day follow‐up period	63 male white New Zealand Rabbits	Animal—63 Rabbits	Anticoagulant Agent: 10% Sodium citrate (1) 900 g 5 min (2) 2000 g 15 min (NR)	60 g 3 min (Sigma 2‐16P Centrifuge, Germany)	(1) Control (*n* = 21) (2) PRP (*n* = 21) (3) i‐PRF (*n* = 21)	*Orthodontic Tooth Movement Evaluation by Digital Orthodontic Gauge (On 7th, 14th, and 28th Day)*: Non‐significant differences in orthodontic movement were found among the three groups on day 7. However, significant differences were observed on days 14 and 28. On day 14, the PRP group had significantly greater orthodontic tooth movement compared to the i‐PRF and control groups, while on day 28, the i‐PRF group showed significantly more movement than the PRP group. *Histological and Histomorphometrical Evaluation by H&E Staining (On 7th, 14th, and 28th Day)*: On day 7, the bone remodeling was not obvious in three groups. On day 14, PRP group showed bone remodeling process with bone deposition on the tension side and bone resorption on the compression side. On 28 day, all three groups showed bone remodeling process with the same process described above.
Tian et al. (2022)^57^	Skin regeneration	To compare the repair effects of i‐PRF prepared by a low‐speed short‐time protocol and PRP prepared by secondary centrifugation method on radiation‐induced skin injury models	24 adult male Sprague–Dawley rats	Animal—Rats	(1) 1000 rpm (123 g) 7 min (2) 3000 rpm (1107 g) 10 min (NR) Activation Agent: 10% Calcium Chloride	700 rpm (60 g) 3 min	(1) 45 Gy (2) 45 Gy + PRP (3) 45 Gy + i‐PRF	*Skin Injury Score by Douglas and Fowler Scoring Method (On 18th, 25th, and 50th Day)*: It was shown that the scores in the i‐PRF and PRP experimental groups were significantly lower than those in the control group. *Blood Perfusion Measurement by Laser Doppler Spectrometer (On 3rd, 8th, 18th, 30th, 50th Day)*: At 4 weeks, the blood perfusion volume reached its peak in each group, with the highest levels observed in the 45 Gy group, averaging (206.01 ± 7.91) PU. This was followed by the 45 Gy + PRP group, averaging (156 ± 6.19) PU, and the lowest was in the 45 Gy + i‐PRF group, averaging (134 ± 7.24) PU. Over time, the blood perfusion volume gradually decreased. *Histological Evaluation by H&E Staining and Immunohistochemical Staining of CD34 and F4/80 (On 50th Day)*: The 45 Gy group exhibited significant loss of epidermis and dermis, with numerous inflammatory cells present, and no angiogenesis was noted. In contrast, the 45 Gy + PRP and 45 Gy + i‐PRF groups did not show significant epidermal and dermal necrosis, displaying significantly less necrosis at the radiation sites. Additionally, CD34 and F4/80 immunohistochemical staining of tissues revealed a higher degree of angiogenesis and a lower degree of cell infiltration in the 45 Gy + PRP and 45 Gy + i‐PRF groups. *Oxidative Stress Measurement*: The 45 Gy group showed the highest levels of malondialdehyde and the lowest levels of superoxide dismutase. In contrast, the use of PRP and i‐PRF resulted in decreased malondialdehyde levels and increased superoxide dismutase levels in the tissues.
Eisa et al. (2024)^47^	Testicular regeneration	To compare the therapeutic effect of PRP and i‐PRF on testicular torsion/detorsion injury in rats	40 mature male Wistar rats	Animal— Rats	Anticoagulant Agent: 3.8% Sodium citrate (1) 300 g 15 min (2) 650 g 15 min (NR) Activation Agent: 10% calcium chloride	700 rpm (60 g) 3 min (NR)	(1) Control negative (2) Control positive (3) PRP (4) i‐PRF	*Semen Evaluation, and Hormonal Assay by ELISA (on 30th Day)*: Semen quality and hormonal assays improved in the PRP and i‐PRF‐treated groups, with i‐PRF showing greater effectiveness. *Oxidative stress‐related Gene Expression by RT‐PCR (on 30th Day)*: High significance of CAT, GPx, SOP, IL‐1β, Caspase‐3 and TNF‐α was reported in treated rats with PRP and i‐PRF with superiority to i‐PRF‐treated rats. *Histological and Histochemical Evaluation by H&E, MT, and PAS Staining (on 30th Day)*: Testicular histoarchitecture was enhanced in the PRP and i‐PRF‐treated rats, with the i‐PRF‐treated rats showing superior results.

Abbreviations: ALP, alkaline phosphatase; Annexin V/PI, annexin V/propidium iodide; A‐PRF, advanced platelet‐rich fibrin; BMP‐2, bone morphogenetic protein‐2; CAT, catalase; CCK‐8, Cell Counting Kit‐8; DAPI, 4′,6‐diamidino‐2‐phenylindole; DCF‐DA, dichlorodihydrofluorescein diacetate; DMP‐1, dentin matrix acidic phosphoprotein‐1; DSPP, dentin sialophosphoprotein; EDTA, ethylenediaminetetraacetic acid; EGF, epidermal growth factor; ELISA, enzyme linked immunosorbent assay; FITC, fluorescein isothiocyanate, glutathione peroxidase; Gy, gray; H & E, hematoxylin and eosin; HA, hyaluronic acid; ICRS, International Cartilage Repair Society; IGF‐I, insulin‐like growth factor‐I; IL‐1β, interleukin‐1β; i‐PRF, injectable platelet‐rich fibrin; L‐PRP, leukocyte‐ and platelet‐rich plasma; MT, Masson's trichrome; MTS, (3‐(4,5‐dimethylthiazol‐2‐yl)‐5‐(3‐carboxymethoxyphenyl)‐2‐(4‐sulfophenyl)‐2H‐tetrazolium); NR, not reported; PAS, periodic acid–Schiff; PDGF, platelet‐derived growth factor; P‐PRP, pure platelet‐rich plasma; PRP, platelet‐rich plasma; PT, pickled titanium; PTGS 2, prostaglandin endoperoxide synthase 2; rpm, revolution per minute; RT‐PCR, reverse transcription polymerase chain reaction; Runx2, Runt‐related transcription factor 2; SLA, sand‐blasted with large grit particles followed by acid‐etching; SOP, superoxide dismutase; TCP, tissue culture plastics; TMJ, temporomandibular joint; TNF‐α, tumor necrosis factor‐α; UV‐A, ultraviolet‐A; VEGF, vascular endothelial growth factor.

**TABLE 3:** Summary of the included clinical studies.

Clinical studies								
Authors (Year)	Category	Aim of study	Study Design	Participants	Preparation method of PRP (Device)	Preparation method of i‐PRF (Device)	Groups	Main Methods and Results
Abo‐Heikal et al. (2023)^48^	Pulp regeneration	To evaluate and compare the completion of root formation and the restoration of pulp sensitivity in traumatized necrotic immature maxillary anterior teeth following using i‐PRF versus PRP regenerative scaffolds	Prospective, parallel group, two arms, randomized clinical trial	23 Patients having 24 immature traumatized necrotic maxillary anterior teeth	Anticoagulant Agent: Di‐potassium EDTA (1) 2400 rpm 10 min (2) 3600 rpm 15 min (NR) Activation Agent: 10% calcium chloride	700 rpm 3 min (E‐4000 table top digital centrifuge)	(1) PRP (*n* = 12) (2) i‐PRF (*n* = 12)	*Root‐related Parameters Evaluation by CBCT (At baseline, and 12th Month)*: There was no statistically significant difference between the two groups in terms of the percentage increase in average root length and the percentage decrease in average root canal diameter at the coronal and middle canal levels. However, the i‐PRF group demonstrated a statistically significant greater percentage decrease in average apical canal diameter compared to the PRP group. *Tooth Sensitivity Evaluation by Electric Pulp Tester (At Baseline, 6th, and 12th Month)*: For the recovery of pulp sensibility, there was no statistically significant difference in the electric pulp tester readings between the two groups after 6 months and 12 months.
Akkas and Esen (2024)^49^	Cartilage regeneration	To evaluate the effect of i‐PRF and PRP on pain and maximum mouth opening to improve the clinical outcomes of arthrocentesis	Retrospective study	116 patients with temporomandibular joint disorder	Anticoagulant Agent: 3.2% Sodium citrate 4000 rpm 10 min (NR)	700 rpm 3 min (NR)	(1) Arthrocentesis (*n* = 35) (2) Arthrocentesis + PRP (*n* = 34) (3) Arthrocentesis + i‐PRF (*n* = 47)	*Pain Evaluation by Visual Analogue Scale (At Baseline, 1st Week, 1st, 3rd, and 6th Month)*: No significant difference were found among the groups in terms of pain. *Maximum Mouth Opening by Analogue Caliper (At Baseline, 1st Week, 1st, 3rd, and 6th Month)*: The PRP group showed superior outcomes in the early postoperative period, while the i‐PRF group demonstrated superior outcomes in the late postoperative period.
Vingender et al. (2023)^55^		To evaluate the effect of repeated HA injection, single PRP and single I‐PRF injection on clinical outcome variables in patients with TMJ internal derangements	Prospective cohort study	68 adult patients with 109 TMJ internal derangements who had failed to respond to nonsurgical treatment	GLO‐PRP separation kit – Finland/Korea (NR)	NR	(1) HA (*n* = 28) (2) PRP (*n* = 21) (3) i‐PRF (*n* = 19)	*Pain Evaluation by Visual Analogue Scale (At Baseline, 6th, and 12th Month)*: HA, PRP, and i‐PRF all resulted in a significant reduction in pain levels during all follow‐up examinations, with no significant difference between the values at the 6‐month and 12‐month follow‐ups. *Maximum Mouth Opening (At Baseline, 6th, and 12th Month)*: A significant difference in mouth opening was noted when comparing the HA, PRP, and i‐PRF groups, with hyaluronic acid showing the greatest improvement. The groups receiving autologous blood substances exhibited similar effects.
Sharma et al. (2023)^54^		To evaluate the efficacy of intra‐articular injections of i‐PRF versus PRP in the management of TMDs	Prospective, randomized clinical trial	14 patients with 28 TMJ internal derangements	Anticoagulant Agent: 3.8% Sodium citrate (1) 1000 rpm 7 min (2) 3000 rpm 10 min (NR)	700 rpm 3 min (NR)	(1) Arthrocentesis + PRP (*n* = 7) (2) Arthrocentesis + i‐PRF (*n* = 7)	*Evaluation of Pain by Visual Analogue Scale, and Maximum Mouth Opening, Lateral/Protrusive Movement, and TMJ Sound by Clinical Examination (At Baseline, 1st, 2nd, 3rd, 4th, 5th, 6th, and 9th Month)*: Pain reduction, maximum mouth opening, lateral/protrusive movement, and joint sounds were significant in both groups, with greater significance observed in the i‐PRF group. *Evaluation of Disk Position and Joint Effusion by MRI (At Baseline and 9th Month)*: Disk position improved toward normal in both groups, showing significant changes at the 9‐month follow‐up, with better results in the i‐PRF group.
Ammar et al. (2024)^50^	Orthodontic tooth movement	To evaluate the potential effect of local injection of PRP and i‐PRF on the rate of orthodontic tooth movement	Parallel group, three arms, randomized clinical trial	60 patients with Class II division 1 malocclusion requiring anterior retraction	Anticoagulant Agent: Acid citrate dextrose‐A Double‐spin process (NR)	700 rpm 3 min (NR)	(1) Control (*n* = 20) (2) PRP (*n* = 20) (3) i‐PRF (*n* = 20)	*Rate of Canine Retraction by Superimposition of 3D Models (At Baseline, 1st, 2nd, 3rd, and 4th Month)*: The rate of canine retraction was faster in the experimental groups. The PRP group exhibited significantly slower movement in the second and fourth months compared to the i‐PRF group, while there were no significant differences between the two experimental groups in the first and third months. *Rate of Canine Rotation, and Molar Anchorage Loss by Superimposition of 3D Models (At Baseline, 1st, 2nd, 3rd, and 4th Month)*: Differences in canine rotation were statistically insignificant among the three groups at all assessment times. However, differences in molar mesial movement among the three groups were statistically significant, with greater molar movement observed in the two experimental groups compared to the control group.
Naji et al. (2022)^53^		To compare the effectiveness of the intra‐ligament i‐PRF and PRP on canine movement rate during its orthodontic retraction	Parallel group, two arm, randomized clinical trial (Split‐mouth design)	40 patients whose all first premolars were indicated for extraction	Anticoagulant Agent: Citrate–Phosphate Dextrose solution with Adenine (1) 1500 rpm 10 min (2) 2800 rpm 5 min (NR)	700 rpm 3 min (NR)	(1) Control 1 (*n* = 20) (2) PRP (*n* = 20) (3) Control 2 (*n* = 20) (4) i‐PRF (*n* = 20)	*Rate of Canine Retraction by Dental Casts (On 21st, 42nd, 63rd, and 84th Day)*: A significantly higher rate of canine movement was noted in the PRP intervention group during the first month, unlike the i‐PRF group when compared to the control side. Additionally, the rate of canine retraction was greater in the PRP group during the third month than in the i‐PRF group. *Rate of Canine Rotation, and Molar Anchorage Loss by Dental Casts, and Canine Inclination by Cephalograms (On 21st, 42nd, 63rd, and 84th Day)*: No statistical differences in canine inclination, rotation, and molar anchorage loss were found except for mandibular canine rotation in the PRP group and maxillary canine rotation between the two groups.
Atsu et al. (2023)^51^	Skin regeneration	To compare PRP and i‐PRF injection treatments for facial skin rejuvenation in terms of efficacy, patient satisfaction, and side effects	Prospective cohort study	55 patients who were admitted to our clinic for facial skin rejuvenation due to cosmetic reasons	PRP T‐Lab kit was used: Anticoagulant Agents: 3.8% Sodium citrate (1) 2000 rpm 2 min (T‐Lab M415P Centrifuge)	PRF T‐Lab kit was used: (1) 2000 rpm 2 min (T‐Lab M415P Centrifuge)	(1) PRP (*n* = 23) (2) i‐PRF (*n* = 32)	*Skin Evaluation by Visioscan® VC‐20 Courage High‐Resolution UVA‐Light Video Camera and SELS software (At Baseline, 1st, 3rd, and 6th Month)*: A significant marginal superiority of i‐PRF over PRP was evident only for certain canthal cosmetic parameters, such as canthal smoothness and wrinkles. However, the two groups did not differ regarding other cosmetic regional parameters. The difference in canthal smoothness was significant at 3 months. *Pain Evaluation by Visual Analogue Scale (Following Injection), Patient Satisfaction by Questions (At 6th Month), and Side Effects by Questions (At Baseline, 1st, 3rd, and 6th Month)*: The two groups did not differ in terms of side effects, pain, and patient satisfaction.
Diab et al. (2022)^52^		To evaluate the efficacy of fluid PRF either alone or combined with needling versus PRP in the treatment of atrophic acne scars	Randomized clinical trial (split‐face design)	30 patients with atrophic acne scars	Anticoagulant Agent: EDTA (1) 900 rpm 5 min (2) 2000 rpm 15 min (NR) Activation Agent: 10% calcium chloride	700 rpm 3 min (NR)	(1) PRP (*n* = 15) (2) PRP + Microneedling (*n* = 15) (3) i‐PRF (*n* = 15) (4) i‐PRF + Microneedling (*n* = 15)	*Therapeutic Response by Goodman and Baron's GSGS (At Baseline and 1st Month)*: The acne scars significantly improved in both sides of face in both groups. *Therapeutic Response by Quartile Grading Scale, and Patient's Satisfaction (At Baseline, and 1st Month)*: Based on the quartile grading scale and patient satisfaction, the therapeutic response was significantly higher in the i‐PRF group compared to the PRP group, whether PRP was used alone or in combination with needling.

Abbreviations: A‐PRF, advanced platelet‐rich fibrin; CAT, catalase; CBCT, cone‐beam computed tomography; EDTA, ethylenediaminetetraacetic acid; GSGS, global scarring grading system; HA, hyaluronic acid; i‐PRF, injectable platelet‐rich fibrin; MRI, magnetic resonance imaging; NR, not reported; PAS, periodic acid–Schiff, PDGF, platelet‐derived growth factor; PRP, platelet‐rich plasma; rpm, revolution per minute; TMJ, temporomandibular joint; UVA, ultraviolet A.

**TABLE 4:** Summary of the outcomes from the included studies, highlighting which APCs demonstrated superior performance in general.

Category	Type of Study	Authors (Year)	More Positive Outcome (PRP or i‐PRF)
Skin regeneration	In vitro studies	Xu et al. (2024)^44^	i‐PRF
		Li et al. (2022)^40^	i‐PRF
		Tian et al. (2022)^57^	i‐PRF
		Wang et al. (2019)^41^	i‐PRF
	In vivo studies	Tian et al. (2022)^57^	i‐PRF
	Clinical studies	Atsu et al. (2023)^51^	ND
		Diab et al. (2022)^52^	i‐PRF
Cartilage regeneration	In vitro studies	Abd El Raouf et al. (2019)^56^	i‐PRF
	In vivo studies	Abd El Raouf et al. (2019)^56^	i‐PRF
	Clinical studies	Akkas and Esen (2024)^49^	ND (PRP (short‐term outcome) i‐PRF (long‐term outcome))
		Vingender et al. (2023)^55^	ND
		Sharma et al. (2023)^54^	i‐PRF
Periodontal regeneration	In vitro studies	Zheng et al. (2020)^45^	i‐PRF
		Miron et al. (2017)^31^	i‐PRF
Soft tissue regeneration around dental implants	In vitro studies	Wang et al. (2017)^42^	i‐PRF
Bone regeneration	In vitro studies	Fernández‐Medina et al. (2019)^37^	i‐PRF
		Wang et al. (2017)^43^	i‐PRF
Orthodontic tooth movement	In vivo studies	Alaa et al. (2023)^46^	ND (PRP (short‐term Outcome) i‐PRF (Long‐term Outcome))
	Clinical studies	Ammar et al. (2024)^50^	i‐PRF
		Naji et al. (2022)^53^	PRP
Antimicrobial activity	In vitro studies	Kour et al. (2018)^39^	ND (PRP for *Aa and* i‐PRF for *Pg*)
		Karde et al. (2017)^38^	i‐PRF
Pulp regeneration	In vitro studies	Chai et al. (2019)^36^	i‐PRF
	Clinical studies	Abo‐Heikal et al. (2023)^48^	ND
Testicular regeneration	In vivo studies	Eisa et al. (2024)^47^	i‐PRF
Total number of positive outcome (%)			Positive Outcome for i‐PRF: 18 (72%) Positive Outcome for PRP: 1 (4%) ND: 6 (24%)

Abbreviations: Aa, *Aggregatibacter actinomycetemcomitans*; i‐PRF, injectable platelet‐rich fibrin; Pg, *Porphyromonas gingivalis*; PRP, platelet‐rich plasma; ND, no difference.

### 3.3 Centrifugation protocol of PRP and i‐PRF

Various methods were employed for the preparation of PRP; some studies utilized commercial kits,^44^, ^51^, ^55^ while others implemented either one‐step^37^, ^49^ or two‐step^31^, ^36^, ^37^, ^38^, ^39^, ^40^, ^41^, ^42^, ^43^, ^45^, ^46^, ^47^, ^48^, ^50^, ^52^, ^53^, ^54^, ^56^, ^57^ centrifugation protocols. However, the most common method across the studies involved centrifugation at 900 *g* for 5 min, followed by a 2000 *g* for 15‐min protocol.^36^, ^41^, ^42^, ^43^, ^45^, ^46^, ^52^, ^56^ Additionally, the anticoagulant agents present in PRP tubes varied among the studies, with sodium citrate^37^, ^38^, ^39^, ^40^, ^46^, ^47^, ^49^, ^51^, ^54^, ^56^ and ethylenediaminetetraacetic acid (EDTA)^36^, ^41^, ^42^, ^43^, ^45^, ^52^ being the most common. Other anticoagulants, such as acid citrate dextrose,^31^ di‐potassium EDTA,^48^ and citrate–phosphate dextrose solution with adenine,^50^ were each used in one study. Additionally, all the studies that referenced activation agents for PRP utilized calcium chloride as the activating agent.^37^, ^40^, ^47^, ^48^, ^52^, ^57^

On the other hand, the preparation methods for i‐PRF were more consistent, with most studies employing a centrifugation speed of 700 rpm (60 *g* for 3 min),^31^, ^36^, ^37^, ^38^, ^39^, ^40^, ^41^, ^42^, ^43^, ^45^, ^46^, ^47^, ^48^, ^49^, ^50^, ^52^, ^53^, ^54^, ^56^, ^57^ and only a few studies that used a commercial kit or did not report its preparation method.^44^, ^51^, ^55^

### 3.4 Platelet counts of PRP and i‐PRF

Three in vitro studies included in this systematic review examined the platelet counts of PRP and i‐PRF.^37^, ^38^, ^57^ In the study by Karde et al.,^38^ the platelet counts per mm^3^ were reported as follows: i‐PRF (1 434 000 ± 75 233), PRP (1 343 000 ± 81 486), PRF (249 000 ± 61 319), and blood (291 000 ± 51 575). The platelet count in i‐PRF was statistically significant compared to the blood, PRP, and PRF, indicating that i‐PRF contained the highest number of platelets among the tested concentrates.^38^ Similarly, Tian et al.^57^ demonstrated that while the platelet counts of both PRP ((1112.67 ± 14.42) × 10^6^/L) and i‐PRF ((1159.67 ± 10.02) × 10^6^/L) were significantly higher than that of whole blood ((235.35 ± 10.54) × 10^6^/L), i‐PRF also contained significantly more platelets than PRP. In another study, Fernández‐Medina et al.^37^ quantified platelet counts of four protocols of platelet concentrates—i‐PRF, advanced platelet‐rich fibrin (A‐PRF), pure platelet‐rich plasma (P‐PRP), and leukocyte‐ and platelet‐rich plasma (L‐PRP)—as well as for whole blood and natural blood clot. In contrast to the two studies mentioned earlier, their findings showed that A‐PRF (307 ± 14 × 10^3^) and i‐PRF (336 ± 18 × 10^3^) had platelet counts comparable to those of whole blood (362 ± 22 × 10^3^) and a natural clot (362 ± 32 × 10^3^). Also, P‐PRP demonstrated^56^ a three‐fold higher platelet recovery (934 ± 32 × 10^3^) compared to whole blood, while L‐PRP exhibited the highest platelet concentration (1820 ± 17 × 10^3^).^37^

### 3.5 Growth factor release profiles of PRP and i‐PRF

There were also three in vitro studies included in this systematic review that assessed the release of growth factors from PRP and i‐PRF.^31^, ^37^, ^57^ Miron et al.^31^ conducted this experiment over a period of 10 days, showing that while PRP had a higher initial release of growth factors, i‐PRF demonstrated significantly greater long‐term release levels of PDGF‐AA, PDGF‐AB, epidermal growth factor (EGF), and insulin‐like growth factor 1 (IGF‐1) for up to 10 days.^31^ In contrast, PRP exhibited elevated levels of TGF‐β1 and VEGF at the 10‐day mark. Corroborating these results, Tian et al.^57^ also demonstrated that after 3 days, PRP exhibited a higher release of TGF‐β1 and VEGF, while i‐PRF showed increased levels of EGF and IGF‐1. A final publication by Fernández‐Medina et al.^37^ also investigated the release of growth factors over a 14‐day period from 4 different platelet concentrates, including A‐PRF, i‐PRF, P‐PRP, and L‐PRP. It was reported that a significant cumulative release of IGF‐I and PDGF‐BB was noted for the A‐PRF and L‐PRP groups at early time points, whereas similar release profiles of BMP‐2 and VEGF were noted in all protocols.^37^

### 3.6 Skin regeneration

The topic of skin regeneration comprised the highest number of studies in this systematic review, with four in vitro studies,^40^, ^41^, ^44^, ^57^ one of which also included in vivo data,^57^ and two clinical studies.^51^, ^52^

#### 3.6.1 In vitro and in vivo studies on skin regeneration

**FIGURE 3:**
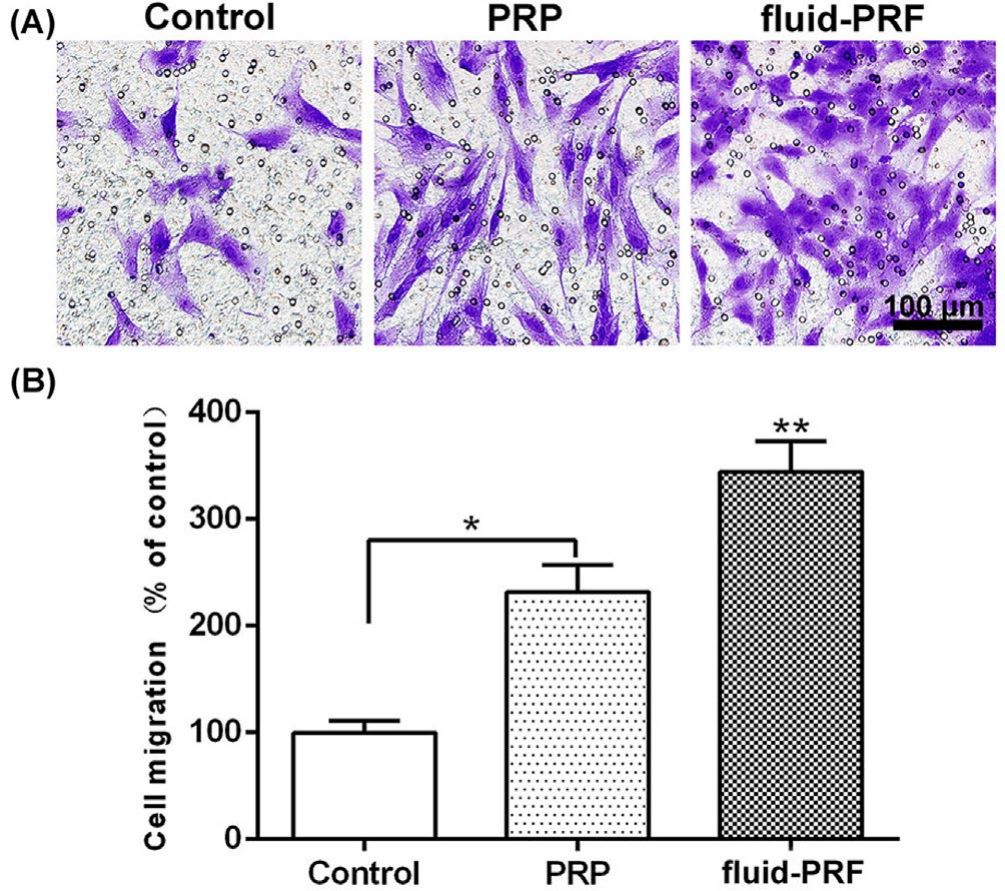
(A, B) Migration assay of human skin fibroblasts cultured with fluid‐PRF and PRP after 24 h. (Scale bars = 100 μm) (*denotes significant difference between two groups *p* < 0.05, ** denotes significantly higher than all other treatment groups *p* < 0.05). Assay performed in triplicate with three independent experiments. Reprinted with permission from Wang et al. 2019.^41^

Three out of four in vitro studies used human dermal fibroblasts,^40^, ^41^, ^57^ whereas one study used both human and mouse dermal papilla cells.^44^ Two of these studies used normal dermal fibroblasts,^41^, ^44^ while the other two investigated radiation‐induced injuries to cells resulting from ultraviolet and X‐ray exposure;^40^, ^57^ one of these studies also included normal cells in addition to the irradiated samples.^57^ All blood samples for these experiments were collected from human participants.^40^, ^41^, ^44^, ^57^ The majority of results in this section demonstrated that i‐PRF produced superior effects compared to PRP in enhancing the proliferative and migratory abilities of dermal‐related cells, as well as inducing a higher expression of regenerative‐ and ECM‐related proteins or genes (e.g., TGF‐β, collagen type 1 (COL1), fibronectin 1 (FN1), alkaline phosphatase (ALP), versican, and β‐catenin; Figure 3).^40^, ^41^, ^44^, ^57^ One study, using immunofluorescent staining, also confirmed that i‐PRF had a significantly greater ability to induce collagen matrix synthesis compared to PRP.^41^ Additionally, two studies indicated that both PRP and i‐PRF could stimulate the TGF‐β/Smad pathway, with i‐PRF exerting a more pronounced effect compared to PRP.^40^, ^44^ It was also shown that i‐PRF is more effective than PRP in reducing the generation of reactive oxygen species (ROS) and cell apoptosis in irradiated dermal cells.^40^, ^57^ In addition to the experiments mentioned above, one study utilized umbilical vein endothelial cells to assess angiogenesis.^57^ The authors demonstrated that after 12 h of culture, vascular endothelial cells exposed to ionizing radiation in the experimental groups had significantly reduced tube‐forming capacity compared to the control group (cells without exposure to ionizing radiation).^57^ In contrast, both PRP and i‐PRF enhanced tube‐forming capacity, even exceeding that of the control group.^57^

**FIGURE 4:**
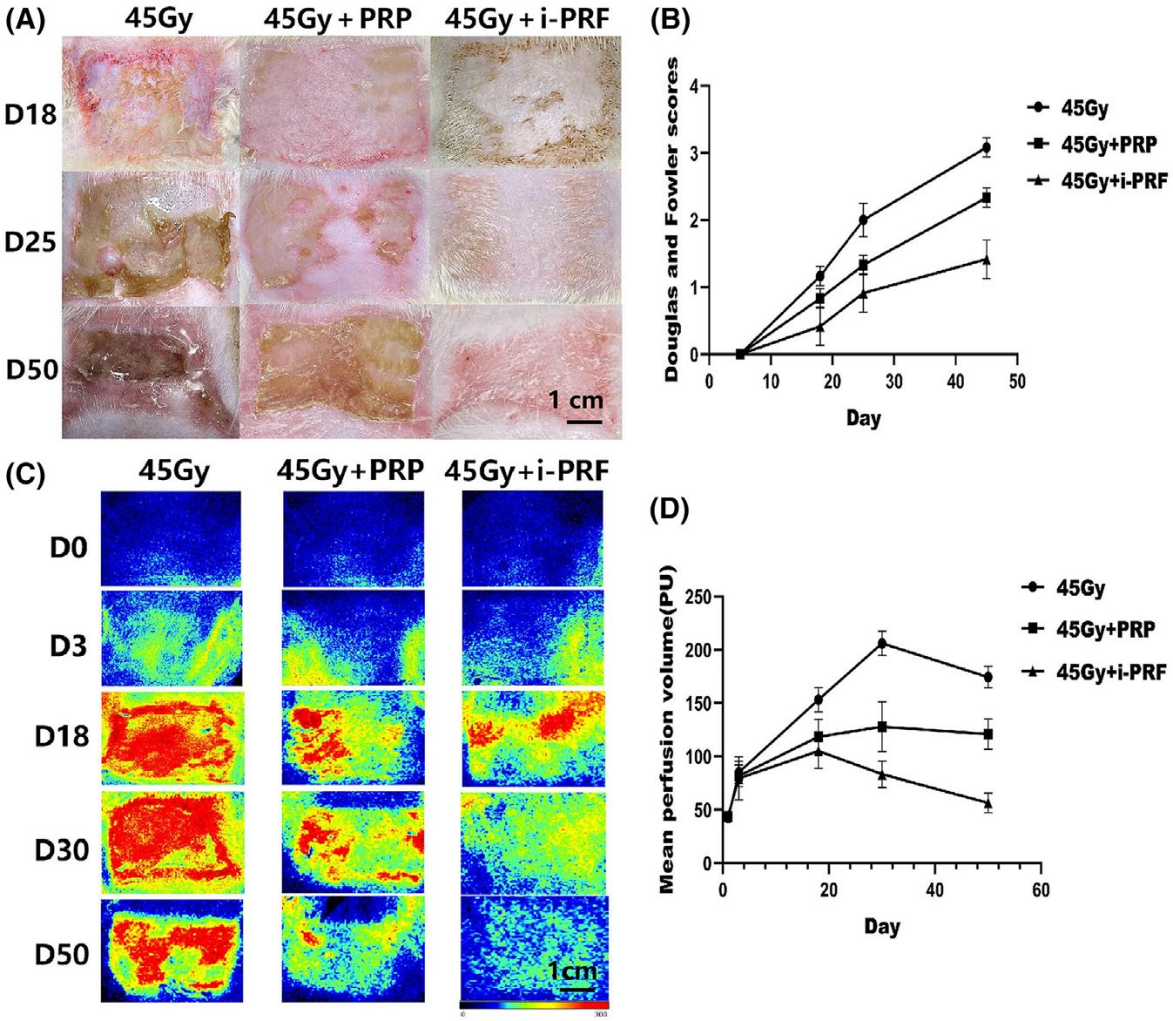
PRP and i‐PRF promoted the healing of radiation‐induced skin injury (RSI). (A) Photographs showing dorsal trauma in the irradiated area of rats. (B) Skin injury scores for dorsal trauma in rats. (C) Laser Doppler perfusion images for each group. (D) Average perfusion measurements across experimental groups. Reprinted with permission from Tian et al. 2023.^57^

Only one in vivo study utilized rats with radiation‐induced skin injuries, which were subsequently treated with PRP and i‐PRF.^57^ The outcomes of this study were evaluated based on skin injury scores, blood perfusion measurements, histological assessments, and oxidative stress evaluations. The results demonstrated that both PRP and i‐PRF could enhance healing, with i‐PRF showing superior results due to its higher concentration of platelets and platelet‐derived growth factors (Figure 4).^57^

#### 3.6.2 Clinical studies on skin regeneration

**FIGURE 5:**
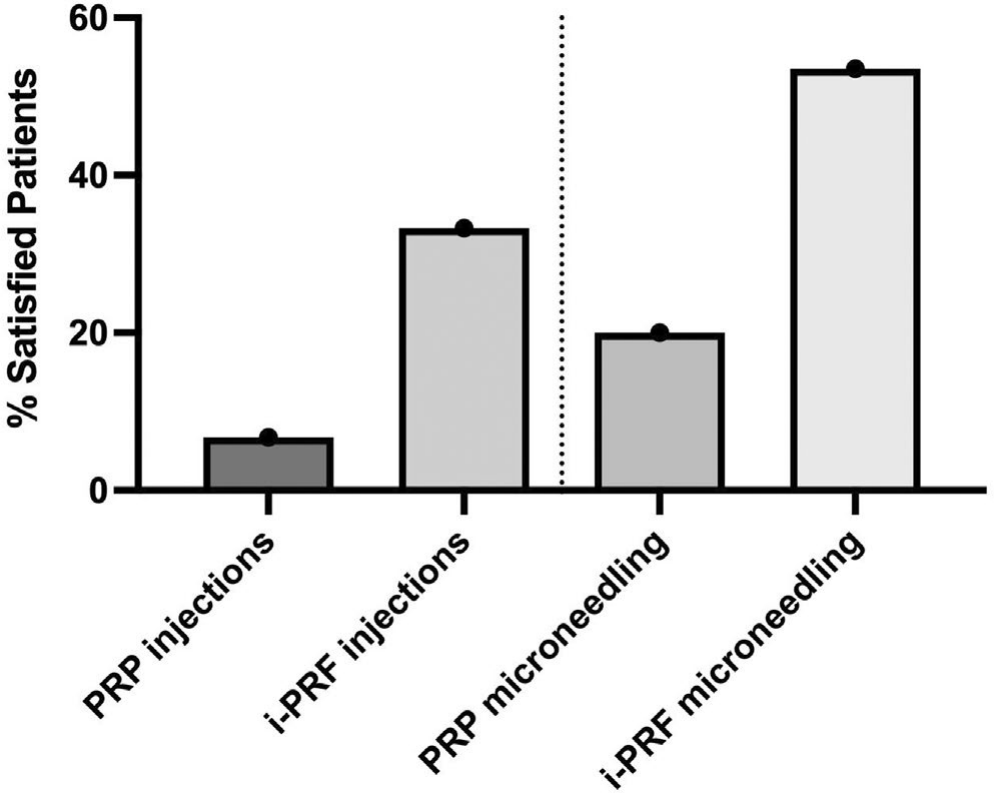
Patients with scars were assigned to one of the following 4 groups. (1) PRP injections, (2) PRF injections, (3) PRP microneedling, and 4) PRF microneedling. Overall, a fold 5 higher “excellent” reported outcomes was found in the PRF injection group when compared to the PRP injection group. Additionally, an over 3‐fold “Excellent” reported score was found in the PRF microneedling group when compared to the PRP microneedling group. In either case, microneedling was better for APC scar treatment when compared to injections. Results derived from Diab et al. 2023.^52^

Both clinical studies were prospective investigations;^51^, ^52^ however, only the study by Diab et al. was a randomized clinical trial.^52^ Atsu et al. focused solely on the application of PRP and i‐PRF for facial skin rejuvenation using intra‐dermal injections,^51^ while Diab et al. utilized PRP and i‐PRF with or without the application of microneedling when compared to intra‐dermal facial injections with PRP/i‐PRF.^52^ In the study by Atsu et al.,^51^ three intra‐dermal injections of either PRP or i‐PRF were administered at one‐month intervals. Patients were then evaluated over a period of 6 months, focusing on clinical parameters, pain intensity, patient satisfaction, and adverse outcomes. The results indicated a significant marginal superiority of i‐PRF over PRP for certain canthal cosmetic parameters, specifically canthal smoothness and wrinkles; however, there were no significant differences between the two groups regarding other cosmetic regional parameters.^51^ Additionally, there were no notable differences between the two groups concerning side effects, pain, and patient satisfaction.^51^ In the other study by Diab et al.,^52^ all patients received four treatment sessions of either PRP or i‐PRF (with or without microneedling) at three‐week intervals, followed by a one‐month follow‐up period. All four groups reported significant improvement in acne scars. According to the quartile grading scale and patient satisfaction assessments, the therapeutic response was significantly higher in the i‐PRF group compared to PRP, whether used alone or in combination with needling.^52^ Additionally, they also stated that the combination of microneedling with i‐PRF or PRP enhanced its efficacy (Figure 5).^52^

### 3.7 Cartilage regeneration

The second most prevalent topic was cartilage regeneration, which included one in vitro,^56^ one in vivo study,^56^ and three clinical studies.^49^, ^54^, ^55^

#### 3.7.1 In vitro and in vivo studies on cartilage regeneration

**FIGURE 6:**
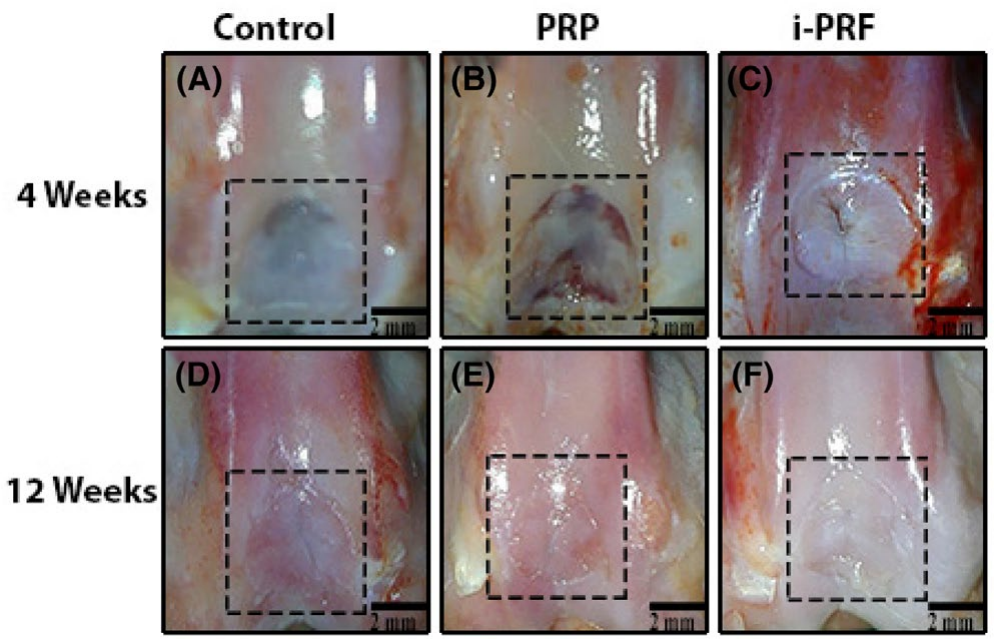
Macroscopic appearance of the osteochondral defects at 4 and 12 weeks post‐operatively in the control group (A and D), PRP group (B and E), and i‐PRF group (C and F). Reprinted with permission from Abd El Raouf et al. (2019).^56^

**FIGURE 7:**
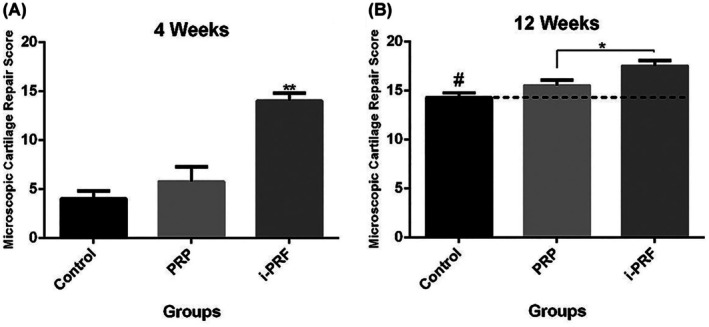
Total ICRS microscopic scores of the regenerated osteochondral defects in the trochlear groove at 4 weeks (A) and 12 weeks (B) post‐operatively. Data are represented as mean ± SD. *Significantly higher than PRP group; *p* < 0.05. **Significantly higher than the control and PRP groups; #Significantly lower than PRP and i‐PRF groups; *p* < 0.05. Reprinted with permission from Abd El Raouf et al. (2019).^56^

In 2019, Abd El Raouf et al.^56^ conducted a study that combined both in vitro and in vivo methods to assess the impact of i‐PRF versus PRP on chondrocytes and the regeneration of osteochondral tissue in a critical‐sized defects in the rabbit knees. For both in vitro and in vivo experiments, blood samples were obtained from rabbits.^56^ In the in vitro section, chondrocytes sourced from the femoral condyle of adult rabbits were used. It was demonstrated that i‐PRF significantly promoted the proliferation and enhanced chondrogenic‐related gene expression (e.g., SOX9, collagen type 2 alpha 1 (COL2A1), and aggrecan (ACAN)) in chondrocytes compared to PRP.^56^ Additionally, in an inflammatory environment induced by interleukin‐1β (IL‐1β), i‐PRF exhibited the highest upregulation of pro‐regenerative gene expression (e.g., SOX9, COL2A1, and ACAN) and downregulation of inflammatory‐related gene expression (e.g., a disintegrin and metalloproteinase with thrombospondin motifs 4 (ADAMTS4) and prostaglandin‐endoperoxide synthase 2 (PTGS2)) in chondrocytes compared to PRP.^56^ In the in vivo animal model, PRP and i‐PRF were used to treat critical‐sized osteochondral defects in the rabbit knees over a 12‐week period, with assessments conducted through macroscopic and microscopic evaluations. By 4 weeks, the macroscopic (International Cartilage Repair Society) ICRS scores for the i‐PRF group showed significant improvement compared to both the PRP and control groups (Figure 6). By 12 weeks, microscopic ICRS scores indicated that the i‐PRF group exhibited a substantial enhancement in cartilage regeneration relative to the PRP group (Figure 7).^56^

#### 3.7.2 Clinical studies on cartilage regeneration

Two of the three clinical studies were prospective,^54^, ^55^ one of which was a randomized clinical trial,^54^ while the third study was retrospective.^49^ All studies involved patients with temporomandibular disorders (TMDs).^49^, ^54^, ^55^ Two of these studies assessed the adjunctive effects of PRP and i‐PRF in conjunction with arthrocentesis treatment,^49^, ^54^ whereas the third study compared the effects of these APCs to hyaluronic acid (HA).^55^ Each study evaluated pain intensity and maximum mouth opening in the patients,^49^, ^54^, ^55^ and one study examined other parameters such as lateral and protrusive movements, TMJ sounds, disk position, and joint effusion using magnetic resonance imaging (MRI).^54^ While Vingender et al. and Akkas and Esen administered a single dose of PRP and i‐PRF,^49^, ^55^ Sharma et al. provided monthly injections of both treatments over a six‐month period.^54^ The results of the included studies were inconsistent; some results reported no significant differences between the PRP and i‐PRF groups,^49^, ^55^ while others demonstrated superior outcomes for i‐PRF.^49^, ^54^ For instance, Sharma et al.^54^ found that arthrocentesis combined with monthly injections of i‐PRF led to significant improvements in pain reduction, maximum mouth opening, lateral and protrusive movements, MRI results, and TMJ sounds, when compared to PRP.^54^ Interestingly, in the study by Akkas and Esen,^49^ which evaluated the adjunctive effects of both PRP and i‐PRF in conjunction with arthrocentesis over a 6‐month period, superior outcomes were noted in the early postoperative phase for the PRP group, while the i‐PRF group demonstrated improved results in the later long‐term postoperative period. However, they found no overall significant difference among the groups (arthrocentesis, arthrocentesis + PRP, and arthrocentesis + i‐PRF) in terms of pain.^49^

### 3.8 Periodontal regeneration

There were only two in vitro studies focused on periodontal regeneration.^31^, ^45^

#### 3.8.1 In Vitro Studies on Periodontal Regeneration

**FIGURE 8:**
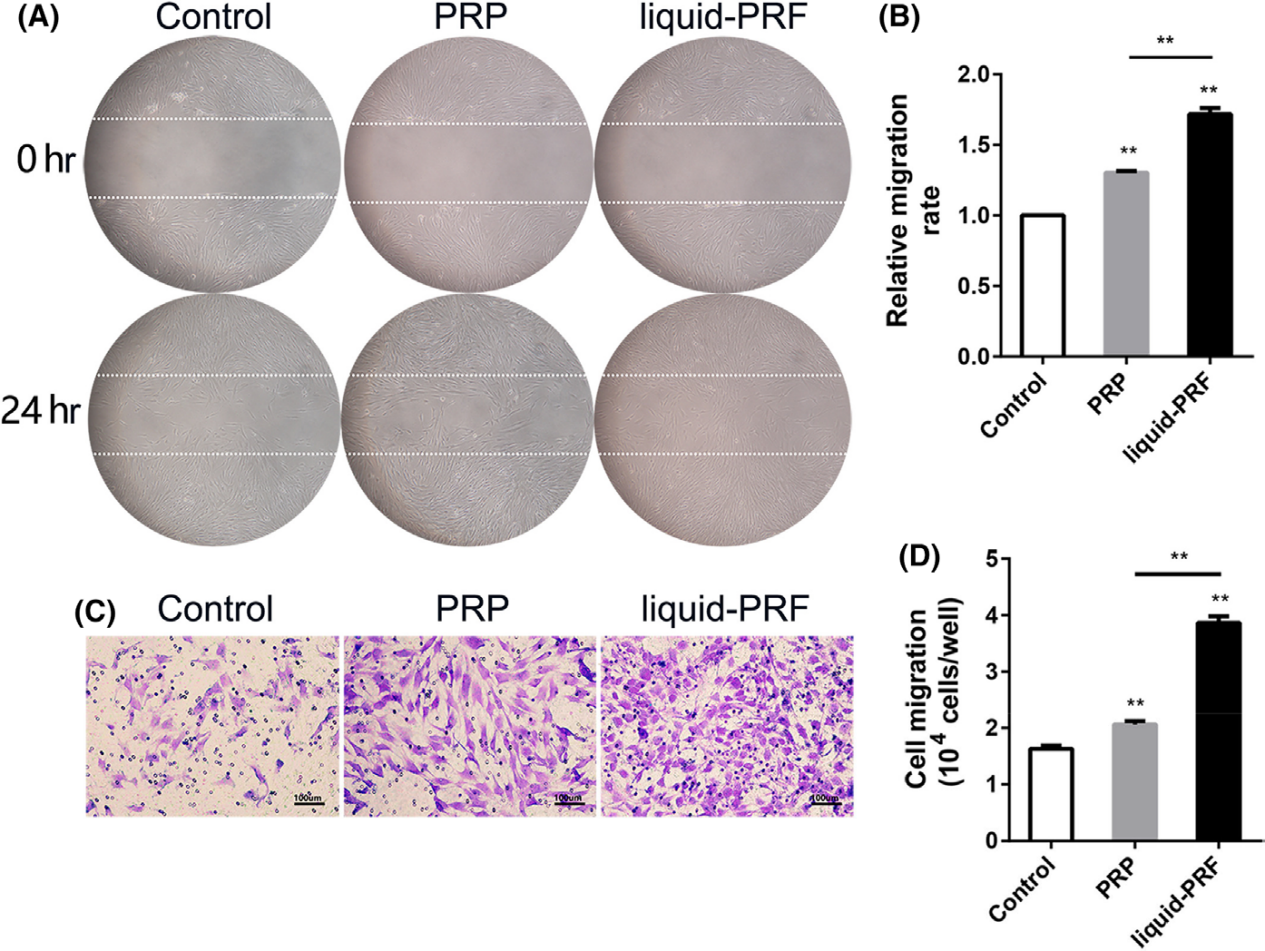
Liquid PRF and PRP accelerated human periodontal ligament cells migration. (A, C) Images showing scratch wound healing and Transwell assays, respectively. (B) Wound scratch area changes after 24 h were used to assess migration ability. (D) Cells in the lower part of the Transwell chamber were quantified after 24 h of culture. Notably, both PRP and liquid PRF significantly promoted cell migration compared to the control group. Data are presented as mean ± SD; **Denotes significant differences between/among groups: *p < 0.05; **p < 0.01; NS, not statistically significant versus the DMEM‐treated group. Reprinted with permission from Zheng et al. (2020).^45^

**FIGURE 9:**
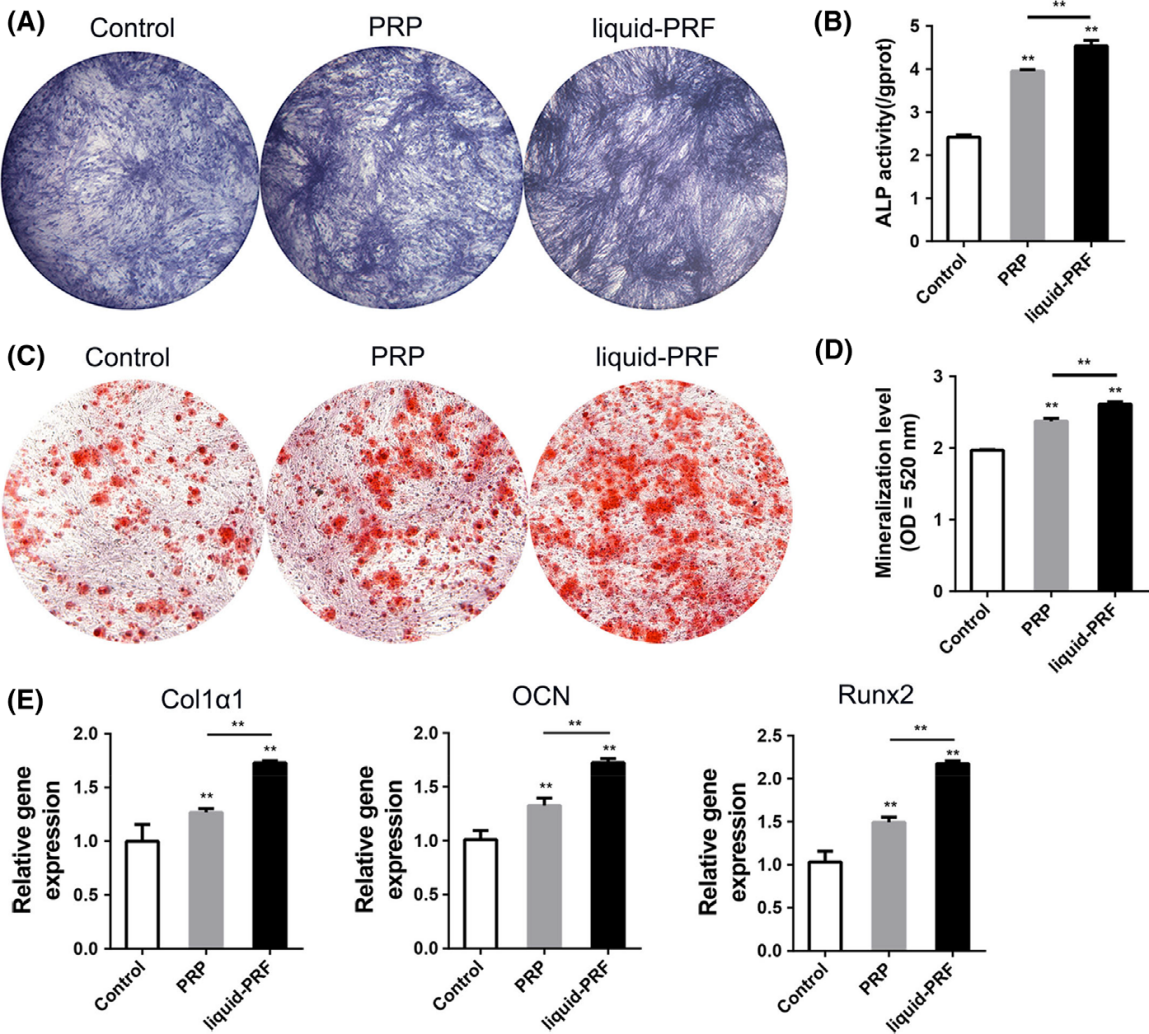
Liquid PRF and PRP promoted osteogenic differentiation of periodontal ligament cells. (A, C) Images of ALP staining and Alizarin Red S staining, respectively. (B) ALP activity assay after 7 days of osteogenic induction. (D) Semi‐quantification of calcium nodules following 14 days of osteogenic induction. (E) Relative expression levels of osteogenic genes Col1a1, OCN, and Runx2 after 14 days of treatment with conditioned medium. Data are presented as mean ± SD; **Indicates significant differences between/among groups: *p < 0.05; **p < 0.01; ns, not statistically significant compared to the control group. Reprinted with permission from Zheng et al. (2020).^45^

One study used human gingival fibroblasts,^31^ while the other used human periodontal ligament cells.^45^ Blood samples for these experiments were all collected from human participants.^31^, ^45^ One study demonstrated that both PRP and i‐PRF exhibited excellent cell viability and biocompatibility.^31^ The proliferative abilities of these autologous concentrates yielded interesting results, with i‐PRF demonstrating significantly superior effects on periodontal ligament cell in one study (Figure 8),^45^ whereas the other study demonstrated that PRP showed improved outcomes for gingival fibroblasts at 3 and 5 days.^31^ Nevertheless, across all studies, i‐PRF consistently achieved significantly superior outcomes compared to PRP in terms of cell migration, mineralization, and the upregulation of osteogenic‐, regeneration‐, and ECM‐related gene expression (e.g., COL1, osteocalcin (OCN), Runt‐related transcription factor 2 (RUNX2), PDGF, TGF‐β; Figures 8 and 9).^31^, ^45^

### 3.9 Soft tissue regeneration around dental implants

There was only one in vitro study that focused on soft tissue regeneration around dental implants.

#### 3.9.1 In vitro studies on soft tissue regeneration around dental implants

In this study, the authors investigated the effects of PRP and i‐PRF on gingival cells cultured on smooth titanium implants (pickled titanium, PT) and roughened titanium implants (sand‐blasted with large grit particles followed by acid‐etching, SLA), compared to control tissue culture plastic (TCP).^42^ Both PRP and i‐PRF demonstrated excellent biocompatibility; however, i‐PRF significantly enhanced cell proliferation, migration, and messenger RNA (mRNA) levels of PDGF, TGF‐β, COL1, and FN in comparison with PRP. Additionally, i‐PRF exhibited the highest fluorescence intensity for COL1 expression across all surfaces.^42^

### 3.10 Bone regeneration

There were only two in vitro studies focused on bone regeneration.^37^, ^43^

#### 3.10.1 In vitro studies on bone regeneration

Both studies employed human osteoblasts.^37^, ^43^ One study collected blood samples obtained from human participants,^43^ while the other used blood obtained from Merino sheep.^37^ While one study compared the effects of i‐PRF versus PRP on osteoblast behavior,^43^ the other examined four different platelet concentrates—A‐PRF, i‐PRF, P‐PRP, and L‐PRP—including two types of PRP, namely P‐PRP and L‐PRP.^37^ In the study by Wang et al.,^43^ both PRP and i‐PRF exhibited high osteoblast viability; however, i‐PRF demonstrated significantly superior proliferative and migratory abilities. Additionally, i‐PRF significantly induced higher ALP activity, alizarin red staining, and increased mRNA levels of ALP, RUNX2, and OCN. Immunofluorescent staining further revealed greater OCN expression in the i‐PRF group compared to the PRP group.^43^ In the other study, Fernández‐Medina et al.^37^ demonstrated that by the 21st day of culture, i‐PRF exhibited superior mineralization properties compared to the other groups, including A‐PRF, P‐PRP, L‐PRP, and blood clot.^37^ The authors also evaluated osteoblast cell metabolic activity over a period of 3 days, finding that L‐PRP and P‐PRP promoted higher metabolic activity compared to the control group.^37^

### 3.11 Orthodontic tooth movement

The topic of OTM included three studies: one in vivo study^46^ and two clinical studies.^50^, ^53^

#### 3.11.1 In vivo studies on orthodontic tooth movement

In the sole study included in this section, Alaa et al.^46^ compared the effects of submucosal injections of i‐PRF and PRP on OTM in a rabbit model over a 28‐day follow‐up period. Sixty‐three rabbits were divided into three equal groups: control, PRP, and i‐PRF. The OTM distance was clinically measured at 7, 14, and 28 days. After each time point, seven rabbits from each group were euthanized for histological evaluation. On day 7, no significant differences in OTM were observed among the three groups. However, significant differences emerged on days 14 and 28. The PRP group exhibited significantly greater OTM than both the i‐PRF and control groups at day 14, while the trend reversed by day 28 with i‐PRF demonstrating the better outcomes. Histological evaluation revealed that on day 7, bone remodeling was not evident in any of the groups. By day 14, the PRP group displayed active bone remodeling, with bone deposition on the tension side and resorption on the compression side. By day 28, all three groups demonstrated a bone remodeling process similar to the description above.^46^

#### 3.11.2 Clinical Studies on Orthodontic Tooth Movement

Both studies were randomized clinical trials,^50^, ^53^ with one employing a split‐mouth design.^53^ The authors investigated the effects of local injections of PRP and i‐PRF on OTM, measuring the rate of canine retraction as the primary outcome and the rates of canine rotation and molar anchorage loss as secondary outcomes.^50^, ^53^ Despite these similarities, some differences in study design were noted. For outcome assessment, Ammar et al. superimposed three‐dimensional (3D) models,^50^ while Naji et al. relied on photographs of dental casts.^53^ Additionally, Naji et al.^53^ evaluated canine inclination as an extra secondary outcome using cephalograms. The duration of the evaluations also varied, with Ammar et al. conducting their study over 4 months,^50^ whereas Naji et al. assessed outcomes over 84 days.^53^ For injection protocols, Ammar et al. administered both intraligamentary and submucosal injections at the start of spring activation and at 21 days,^50^ while Naji et al. used only intraligamentary injections given immediately before canine retraction and at 8 weeks.^53^ The results between these two studies were inconsistent. Ammar et al. found that the rate of canine retraction was faster in the i‐PRF group during the second and fourth months, while no significant differences were observed between the two groups in the first and third months.^50^ Conversely, Naji et al. reported a significantly higher rate of canine movement in the PRP group compared to the control side during the first month, which was not observed in the i‐PRF group.^53^ Additionally, the canine retraction rate remained higher in the PRP group than in the i‐PRF group during the third month.^53^ For secondary outcomes, Ammar et al. found no statistically significant differences in canine rotation across the three groups at any time points. However, molar mesial movement was significantly greater in both experimental groups compared to the control.^50^ Naji et al. similarly observed no significant differences in canine inclination, rotation, or molar anchorage loss, except for mandibular canine rotation in the PRP group and maxillary canine rotation between the PRP and i‐PRF groups.^53^ Overall, there was no superiority detected for either PRP or i‐PRF in the clinical studies.

### 3.12 Antimicrobial activity

Only two in vitro studies compared the antimicrobial activities of PRP and i‐PRF.^38^, ^39^

#### 3.12.1 In vitro studies on antimicrobial activity

Both studies compared the antimicrobial activity of PRP, PRF, and i‐PRF.^38^, ^39^ In both studies, blood samples were collected from human patients;^38^, ^39^ specifically, one study sourced samples from healthy individuals,^39^ while the other study collected samples from patients with chronic generalized marginal gingivitis.^38^ One study utilized *Porphyromonas gingivalis* (Pg) and *Aggregatibacter actinomycetemcomitans* (Aa) as bacterial sources,^39^ while the other study derived bacteria from supragingival plaque samples obtained from individuals who volunteered for blood donation.^38^ Both studies employed disk diffusion tests to evaluate the antimicrobial activity of these APCs.^38^, ^39^ In the first study, i‐PRF produced the largest zone of inhibition against Pg, significantly wider than that of PRF and PRP.^39^ In contrast, for the Aa group, PRP displayed a larger zone of inhibition than both PRF and i‐PRF.^39^ In the other study involving supragingival plaques, the mean zones of inhibition for i‐PRF and PRF were statistically significant. Although PRP displayed a noticeable zone of inhibition, this finding did not reach statistical significance.^38^

### 3.13 Pulp regeneration

One in vitro study and one clinical study were identified that focused on pulp regeneration.^36^

#### 3.13.1 In vitro studies on pulp regeneration

**FIGURE 10:**
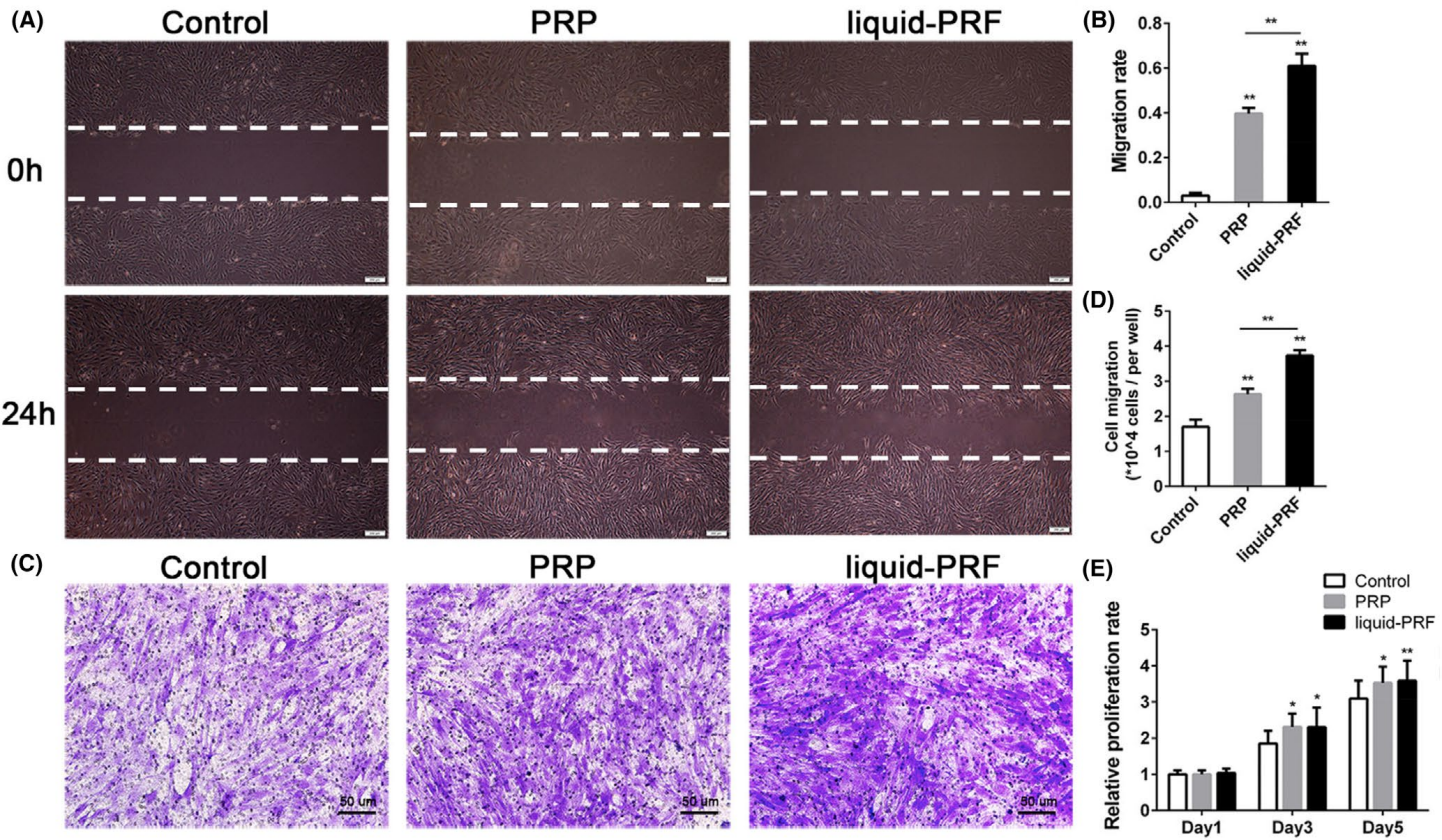
The migration and proliferation of human dental pulp cells cultured with PRP or liquid PRF were assessed. (A) Images from a scratch wound healing assay. (B) Migration rates for each group were calculated based on area coverage after 24 h. (C) Images from a Transwell migration assay. (D) The number of migrated cells (10^4^ per well) in the Transwell migration assay was analyzed after 12 h of culture. PRP and liquid PRF significantly enhanced cell migration compared to the control. (E) A Cell Counting Kit‐8 assay was performed to evaluate the proliferation rate of PRP‐ and liquid PRF‐treated cells over a 5‐day period, showing a significant increase in cell numbers compared to the control. Error bars represent mean ± standard deviation. Significant differences are indicated: *p < 0.05, **p < 0.01 compared to the control. ns, not statistically significant versus the control group. Reprinted with permission from Chai et al. (2019).^36^

**FIGURE 11:**
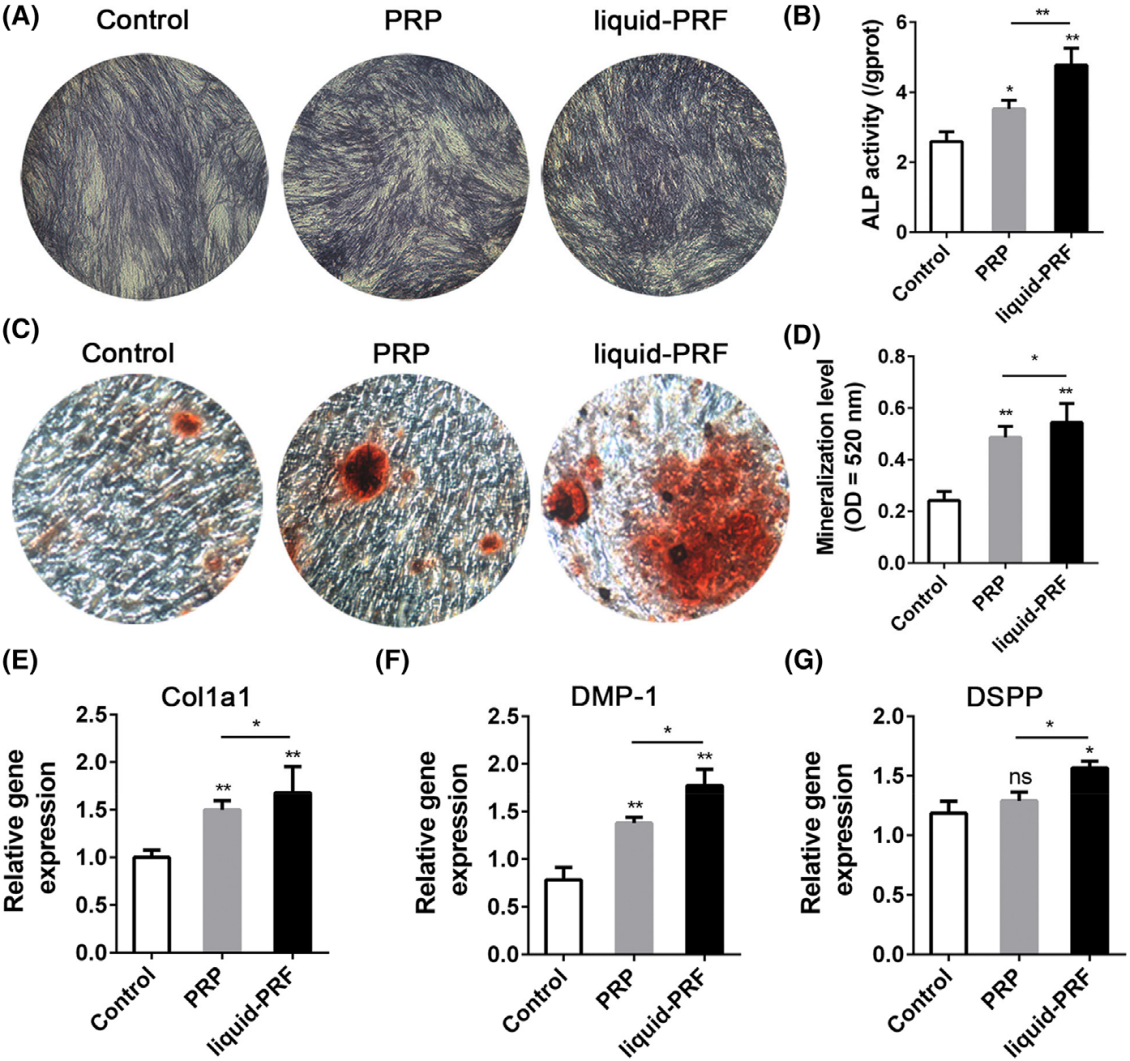
The effect of PRP and liquid PRF on odontoblast differentiation of human dental pulp cells was evaluated. (A, B) The impact of PRP and liquid PRF on ALP activity was assessed through (A) ALP staining and (B) an ALP activity assay. (C, D) Alizarin Red S staining revealed mineralized nodules in each group after 14 days of induction; the right panel shows the semi‐quantification of mineralization levels. (E–G) Relative gene expression levels of (E) Col1a1, (F) DMP‐1, and (G) DSPP were analyzed after 14 days of culture with PRP or liquid PRF. Error bars represent mean ± SD. Significant differences are indicated: *p < 0.05, **p < 0.01; ns, not statistically significant compared to the control group. Reprinted with permission from Chai et al. (2019).^36^

In this study, Chai et al.^36^ investigated the cellular regenerative activity of human dental pulp cells (hDPCs) cultured with either i‐PRF or PRP, with blood samples obtained from individual patients. The results demonstrated that i‐PRF significantly enhanced the proliferation and migration of hDPCs, as well as increased ALP activity, alizarin red staining, and the expression of odontogenic‐related genes (e.g., COL1a1, dentin sialophosphoprotein (DSPP), and dentin matrix acidic phosphoprotein 1 (DMP‐1)) compared to PRP (Figures 10 and 11).^36^

#### Clinical studies on pulp regeneration

In this clinical study, Abo‐Heikal et al.^48^ compared the regenerative potential of i‐PRF and PRP for pulp regeneration. They enrolled 23 patients with a total of 24 immature, traumatized, necrotic maxillary anterior teeth. During the first appointment, the canals were irrigated with 1.5% sodium hypochlorite and treated with calcium hydroxide medication; the APCs were then applied into the canals at the second appointment. Outcomes were evaluated at 6‐ and 12‐months post‐treatment using clinical and radiographic examinations.^48^ They found no statistically significant difference between the two groups in terms of root length increase, coronal and middle canal diameter reduction, or response to the electric pulp tester. However, the i‐PRF group demonstrated a significantly greater decrease in apical canal diameter compared to the PRP group.^48^

### 3.14 Testicular regeneration

There was only one in vivo study that focused on testicular regeneration.^47^

#### 3.14.1 In vivo studies on testicular regeneration

In this study, which was conducted by Eisa et al. in 2024,^47^ they compared the therapeutic effect of PRP and i‐PRF on testicular torsion/detorsion injury in rats. The authors twisted the right testis in experimental groups (control positive, PRP, and i‐PRF groups) 1080° clockwise for 3 h. Following detorsion, 10 μL of PRP or i‐PRF was injected intra‐testicular 3 h later in the PRP and i‐PRF groups, respectively. After 30 days post‐surgery, improvements in semen quality and hormonal assays were observed in both the PRP and i‐PRF treatment groups, with i‐PRF showing superior results. Treated rats exhibited significant increases in catalase (CAT), glutathione peroxidase (GPx), superoxide dismutase (SOP), IL‐1β, caspase‐3, and tumor necrosis factor‐α (TNF‐α) levels, with i‐PRF‐treated rats showing even greater improvements. Histological evaluations of testicular architecture also revealed enhancements in both PRP and i‐PRF‐treated rats, with i‐PRF‐treated rats demonstrating superior histological outcomes.^47^

## 4 DISCUSSION

This systematic review, for the first time, evaluated the comparative effects of i‐PRF and PRP across various fields of regenerative medicine. We first examined the preparation protocols for these platelet concentrates, along with their platelet counts and growth factor profiles. Subsequently, we compared their effectiveness in regenerating skin, cartilage, periodontal tissue, bone, pulp, testicular tissue, and soft tissue around dental implants, along with their antibacterial properties and influence on OTM.

In terms of preparation protocols, i‐PRF followed a more standardized centrifugation protocol (700 rpm (60 g) for 3 min) across the majority of studies, whereas PRP preparation was much more variable, involving diverse centrifugation speeds and durations. Recently, Gruber et al. compared and contrasted the heterogenicity of PRP preparations (1 vs. 2 steps, tubes, anticoagulation, etc.) and noted the complexity of PRP preparation.^58^ This variability in PRP preparation could potentially account for some of the differences regarding the presented in vitro, in vivo, or clinical outcomes, as the concentration of platelets and composition of growth factors may be affected by these parameters. Furthermore, one important aspect regarding PRP preparation has been whether or not activators were utilized. It was also noted that many of the studies were derived from in vitro experiments with limitations including their impact in a clinical setting with high donor variance, small sample size, and minimal statistical power reported.

In terms of platelet counts, i‐PRF generally yielded significantly higher platelet concentrations than PRP.^31^, ^57^ However, one study that compared i‐PRF with two modified types of PRP, namely P‐PRP and L‐PRP, reported the opposite results, indicating that P‐PRP and L‐PRP produced higher platelet concentrations.^37^ In terms of growth factor release profiles, PRP demonstrated higher initial release of growth factors, while i‐PRF exhibited a more sustained and long‐term release profile.^31^ Similar outcomes were also observed in another study comparing the growth factor release of PRP to that of solid PRF.^15^ Notably, i‐PRF showed significantly greater release levels of PDGF‐AA, PDGF‐AB, EGF, and IGF‐1, whereas PRP was more associated with elevated levels of TGF‐β1 and VEGF.^31^, ^57^ However, these findings were inconsistent with those of the study by Fernández‐Medina, which used two modifications of PRP (P‐PRP and L‐PRP).^37^ These discrepancies in platelet counts and growth factor release profiles may be attributed to the varying centrifugation parameters used for PRP preparation in different studies, as mentioned above.

In the in vitro experiments, it was found that while both i‐PRF and PRP enhanced tissue regeneration, in most of the studies, i‐PRF was found to be more effective than PRP in promoting the proliferation, migration, mineralization, and gene expression of regeneration‐related factors in cells involved in the regeneration of skin,^40^, ^41^, ^44^, ^57^ cartilage,^56^ periodontal,^31^, ^41^ bone,^43^ soft tissues around dental implants,^42^ and pulp tissues.^36^ These enhanced outcomes of i‐PRF may be attributed to its higher platelet concentrations and the sustained release of growth factors over time, which creates a more favorable environment for cell proliferation, migration, differentiation, and matrix remodeling. Additionally, the absence of anticoagulants during i‐PRF preparation may further contribute to its effectiveness since clotting is one of the most important initial steps toward healing.^59^

In other in vitro studies investigating antimicrobial activity, i‐PRF demonstrated superior outcomes in inhibiting bacterial growth from supragingival calculus and *Pg*,^38^, ^39^ while PRP showed a more pronounced effect on *Aa*.^39^ The antibacterial effect of i‐PRF may be attributed to its higher leukocyte concentrations,^39^ resulting from its lower centrifugation time and speed.^60^ However, it is important to consider the potential antibacterial effects of anticoagulants, such as sodium citrate which was used in the preparation of PRP in that study. Sodium citrate has been shown to exhibit antibacterial activity against *Streptococcus pneumoniae* and various oral bacteria.^61^

Most in vivo experimental results also favored i‐PRF. In vivo studies assessing the regeneration of skin, cartilage, and testicular tissues demonstrated that both PRP and i‐PRF enhanced healing, with i‐PRF showing significantly superior results in all studies.^47^, ^56^, ^57^ Additionally, an in vivo study on OTM showed that while submucosal injection of PRP accelerates tooth movement significantly more than i‐PRF at an early time point (14 days), i‐PRF reversed that trend at later time points (28 days), indicating a more pronounced long‐term effect of i‐PRF compared to PRP.^46^ This may be explained by i‐PRF's sustained release of growth factors over an extended period of time, in contrast to PRP's more immediate but shorter‐lasting release.^31^

In the eight clinical studies, the results were more heterogeneous, with i‐PRF often yielding superior outcomes. In clinical studies focused on skin regeneration, i‐PRF outperformed PRP in enhancing some specific cosmetic parameters.^51^ Furthermore, intradermal injections of i‐PRF produced significantly better results than intradermal injections with PRP; the same was true when i‐PRF was micro‐needled into the skin compared to PRP in treating patients with atrophic acne scars.^52^ This could be explained by the fact that in in vitro studies, i‐PRF was shown to significantly enhance the proliferation and migration of dermal cells and to upregulate COL1 expression, which is essential for tissue regeneration and wound healing, when compared to PRP.^40^, ^41^, ^44^ Additionally, findings indicated that i‐PRF had a more pronounced effect on the TGF‐β/Smad pathway than PRP, which can influence various cellular processes critical for promoting skin regeneration.^40^, ^44^ This finding may contribute to the superior results observed in clinical applications, especially in skin rejuvenation and scar treatment.

For pulp regeneration, i‐PRF was able to significantly regenerate the apical portion of the root by decreasing the apical canal diameter in comparison with PRP in immature traumatized teeth.^48^ However, it did not demonstrate any superiority regarding other parameters such as root length increase, coronal and middle canal diameter reduction, or response to the electric pulp tester.^48^ It has been demonstrated that i‐PRF induces higher expression of DSPP and DMP‐1 than PRP, both of which are essential for mineralization and dentin formation.^36^ Additionally, the lack of significance between the treatment groups for increased root length may be attributed to the fact that this increase often occurs 18–24 months post‐treatment^62^; however, the follow‐up time point of this study was only 12 months.^48^

In clinical studies examining the treatment of TMDs through cartilage regeneration, although one study found no significant difference between i‐PRF and PRP across various outcomes, such as pain intensity and maximum mouth opening,^55^ another study indicated that while superior outcomes in maximum mouth opening were observed in the early postoperative period for the PRP group, the i‐PRF group demonstrated better results in the late postoperative period.^49^ Additionally, a final study also supported the use of i‐PRF, showing that it significantly improved TMD outcomes compared to PRP.^54^ The inconsistency may stem from the frequency of injections, as Sharma et al. administered six repetitions of APCs injections,^54^ while the other two studies used only a single dose.^49^, ^55^ Even though i‐PRF has a longer release profile for growth factors than PRP, it can only release them over a period of 10–14 days.^63^ Therefore, for applications that require prolonged growth factor activity, it may be beneficial to repeat this treatment. For OTM, the clinical studies also presented mixed results. While Naji et al.^53^ concluded that PRP was likely more effective than i‐PRF in accelerating canine movement, Ammar et al.^50^ found both PRP and i‐PRF to be effective, with i‐PRF showing longer‐lasting effects. The inconsistent results between these two studies may also be attributed to differences in injection sites (intraligamental or submucosal), as well as variations in the timing and frequency of APCs administration.^64^ Current evidence also suggests that boosting injections of APC may help maintain and enhance the rate of OTM.^64^

In total, 72% of studies found better outcomes for the use of i‐PRF when compared to PRP, and 24% found no difference between the two APCs (Table 4). Only one study investigating OTM found better outcomes for PRP, and this may be attributed to the fact that the anticoagulants in PRP may have facilitated faster bone resorption, or that the quick burst/release of growth factors from PRP may have sped up osteoclastic activity.^65^, ^66^ Noteworthy, the majority of systematic reviews on PRF have found that PRF is far superior for soft tissue wound healing when compared to hard tissues.^7^, ^27^, ^67^, ^68^, ^69^, ^70^ Additionally, it was reported that the shorter, one‐step centrifugation process for i‐PRF, compared to the longer one‐ or two‐step process for PRP, makes i‐PRF more practical for clinical applications.

One important point to consider was that there was very little discussion in all studies regarding the effects of protocols on platelet and/or leukocyte concentrations. In a recent publication by our group, an evaluation of 24 protocols investigating the effects of RCF values and time on the production of liquid PRF found marked differences in the concentration of cells.^71^ This led to significant development in protocols to better optimize the fabrication of PRF that includes horizontal centrifugation^72^, ^73^, ^74^, ^75^ as well as novel protocols termed concentrated‐PRF (C‐PRF).^13^, ^76^, ^77^ Furthermore, very little discussion was placed on the actual tube types utilized in each study, which not only focused on the anticoagulant and their associated concentrations utilized but also the hydrophobic/hydrophilic properties of the tubes, as well as their surface roughness.^22^, ^78^, ^79^ It has been shown that each of these factors can affect the ability of cells to bind to their surfaces and either promoting or preventing cells from separating into their appropriate layers following centrifugation.^22^, ^78^, ^79^

## 5 CONCLUSION AND CLINICAL RECOMMENDATIONS

In conclusion, it was generally observed in the majority of studies that i‐PRF demonstrated superior regenerative capacity in various tissue types, particularly in applications requiring sustained and prolonged growth factor release. However, PRP may offer some advantages in applications where a rapid but short‐term release of growth factors is desired. The findings may suggest that i‐PRF may have broader applications due to its consistent regenerative potential, yet further clinical trials are needed to fully elucidate the specific contexts where each treatment modality as well as APC protocol may offer the greatest benefit.

Despite the limited evidence in the literature and the need for further clinical trials, the following recommendations may help clinicians select the appropriate APC based on specific clinical indications and desired biological outcomes:

- i‐PRF is recommended for procedures requiring prolonged regenerative effects, such as bone and periodontal regenerative procedures, soft tissue healing around dental implants, orthopedic cartilage regeneration, and facial esthetic procedures.
- As an antimicrobial agent, the incorporation of leukocytes into i‐PRF has been found to favor better antimicrobial activity.
- i‐PRF can be considered a viable scaffold for clinical use in regenerative endodontic therapy.
- The use of i‐PRF as a therapeutic modality for the regeneration of testicular damage following severe testicular torsion could also be recommended.

## AUTHOR CONTRIBUTIONS

All authors made substantial contributions to the conception and design of the manuscript. NF and MAA performed the literature search. All authors drafted the work and revised it critically for important intellectual content, agreed to be accountable for all aspects of the study design and its content, and approved the final submitted version.

## CONFLICT OF INTEREST STATEMENT

Richard J. Miron is the founder of Miron Research and Development in Dentistry LLC that holds intellectual property on the production of PRF. All other authors declare that they have no competing interest.

## ETHICS STATEMENT

No ethics approval was required for this study since it was a systematic review.

## INFORMED CONSENT

No informed consent was required.

## Data Availability

The data that supports the findings of this study are available in the supplementary material and via the corresponding author of this article.
